# Identification of iron and zinc responsive genes in pearl millet using genome-wide RNA-sequencing approach

**DOI:** 10.3389/fnut.2022.884381

**Published:** 2022-11-09

**Authors:** Chengeshpur Anjali Goud, Vanisri Satturu, Renuka Malipatil, Aswini Viswanath, Janani Semalaiyappan, Himabindu Kudapa, Santosha Rathod, Abhishek Rathore, Mahalingam Govindaraj, Nepolean Thirunavukkarasu

**Affiliations:** ^1^Institute of Biotechnology, Professor Jayashankar Telangana State Agricultural University, Hyderabad, India; ^2^Genomics and Molecular Breeding Lab, ICAR-Indian Institute of Millets Research, Hyderabad, India; ^3^International Crops Research Institute for the Semi-Arid Tropics (ICRISAT), Hyderabad, India; ^4^Agricultural Statistics, ICAR-Indian Institute of Rice Research, Hyderabad, India; ^5^Excellence in Breeding Platform, The International Maize and Wheat Improvement Center (CIMMYT), Texcoco, Mexico; ^6^HarvestPlus, Alliance of Bioversity International and the International Center for Tropical Agriculture (CIAT), Cali, Colombia

**Keywords:** malnutrition, biofortification, iron, zinc, genes, transcriptome

## Abstract

Pearl millet (*Pennisetum glaucum* L.), an important source of iron (Fe) and zinc (Zn) for millions of families in dryland tropics, helps in eradicating micronutrient malnutrition. The crop is rich in Fe and Zn, therefore, identification of the key genes operating the mineral pathways is an important step to accelerate the development of biofortified cultivars. In a first-of-its-kind experiment, leaf and root samples of a pearl millet inbred ICMB 1505 were exposed to combinations of Fe and Zn stress conditions using the hydroponics method, and a whole-genome transcriptome assay was carried out to characterize the differentially expressed genes (DEGs) and pathways. A total of 37,093 DEGs under different combinations of stress conditions were identified, of which, 7,023 and 9,996 DEGs were reported in the leaf and root stress treatments, respectively. Among the 10,194 unique DEGs, 8,605 were annotated to cellular, biological, and molecular functions and 458 DEGs were assigned to 39 pathways. The results revealed the expression of major genes related to the mugineic acid pathway, phytohormones, chlorophyll biosynthesis, photosynthesis, and carbohydrate metabolism during Fe and Zn stress. The cross-talks between the Fe and Zn provided information on their dual and opposite regulation of key uptake and transporter genes under Fe and Zn deficiency. SNP haplotypes in rice, maize, sorghum, and foxtail millet as well as in Arabidopsis using pearl millet Fe and Zn responsive genes could be used for designing the markers in staple crops. Our results will assist in developing Fe and Zn-efficient pearl millet varieties in biofortification breeding programs and precision delivery mechanisms to ameliorate malnutrition in South Asia and Sub-Saharan Africa.

## Introduction

Pearl millet is a high-energy cereal with high protein, and high dietary fiber (1), free of gluten (2) with higher amounts of iron (Fe) and zinc (Zn) accounting for up to 40%. It is the economical source of micronutrients for poor people, who suffer from micronutrient deficiencies (3). Owing to breeding for yield and yield-contributing traits over the decades, less emphasis has been given to breeding nutritional traits (4, 5). This signifies the importance of improving the essential grain micronutrients (Fe and Zn) in future pearl millet varieties besides core breeding traits.

Malnutrition refers to the inadequate nutrient supply and/or inefficient uptake resulting from an imbalance of essential nutrients in the regular diet of an individual or population. Globally, 144 million children under 5 years are stunted and 45% of child deaths are associated with malnutrition.^1^ Worldwide, non-pregnant women (33%), pregnant women (40%), and children (42%) are reported with Fe deficiency (see text footnote 1) resulting in low body weight of the child, and maternal mortality affecting both mother and infants (6). Fe deficiency causes improper functioning of the immune system, and poor health, and may reduce the working capacity of a person (7). Zn deficiency causes stunted growth, and improper neural development, and is highly susceptible to disease attack. It is reported to be a major cause of respiratory infections leading to high infant mortality (8).

Compared to sorghum (26–60 mg kg^–1^ Fe and 21–57 mg kg^–1^ Zn) (9), maize (18.9–47.6 mg kg^–1^ Fe and 5.4–30.8 mg kg^–1^ Zn) (10), and foxtail millet (36.9–75.1 mg kg^–1^ Fe and 45.4–57.1 mg kg^–1^ Zn) (11), the pearl millet possesses high grain Fe (30–140 mg kg^–1^) and Zn (20–90 mg kg^–1^) contents in the germplasm (5, 12). So, pearl millet has gained importance concerning available wider genetic variability for Fe and Zn contents in germplasm and its ability to grow in harsh environmental conditions (13). Several studies reported a positive correlation between the densities of Fe and Zn, which helps in the selection of both Fe and Zn micronutrients simultaneously (14), and are assumed to be involved or connected in physiological mechanisms for their uptake and translocation into the seed (15, 16). To combat the micronutrient-based hidden hunger, biofortification would be an efficient and cost-effective method (3) to enhance the nutrient contents of crops through breeding techniques (17). Pearl millet was one of the crops included in the Biofortification Challenge Program (BCP), a micronutrient project in 2002 under the HarvestPlus program of the CGIAR for decreasing Fe and Zn deficiencies (18). To date, HarvestPlus supported the pearl millet biofortification program at ICRISAT for the development of several high Fe and Zn lines and cultivars in association with NARS (5).

Understanding the functional genomics of Fe and Zn homeostasis and their uptake and transport mechanism will assist in the biofortification of Fe and Zn content in crops. Transcriptomic studies on functional characterization of differentially expressed genes (DEGs) and their dynamic role in Fe and Zn uptake in deficiency, which helps in genetic biofortification of Fe and Zn, have been carried out in rice (19), maize (20), Arabidopsis (21), and several other crops. The above studies reported the importance of genes related to the mugineic acid (MA) pathway, plant hormones, and carbohydrate metabolism in nutrient uptake and the negative effect on photosynthesis-related genes under deficient conditions.

So far, there has been no research carried out on pearl millet to understand the functional mechanisms of Fe and Zn homeostasis through a genome-wide transcriptome approach although the crop is known for its rich iron and zinc content. Hence, this study was constituted to understand the gene expression of pearl millet shoot and root under Fe and Zn stress through the RNA-Seq approach to identify the DEGs, and gene regulatory networks under mineral stress conditions and identify the genes involved in Fe and Zn homeostasis.

## Materials and methods

### Experiment material and growth conditions

Seeds of the biofortified pearl millet inbred ICMB 1505 (a high Fe inbred with 110 ppm Fe and 55 ppm Zn) using 1% sodium hypochlorite solution were surface sterilized for 5 min and rinsed 5–6 times with MilliQ water. The seeds were placed on the germination sheets soaked with MilliQ water and allowed to germinate in the darkroom at 25°C temperature.

Three nutrient stress treatments (+Fe−Zn, −Fe+Zn, and −Fe−Zn) and one control (+Fe+Zn) solutions were prepared to understand the stress response of the root and leaf. The control nutrient solution (+Fe+Zn) contained 0.7 mM K_2_SO_4_, 0.1 mM KCL, 2.0 mM Ca (NO_3_)_2_, 0.1 mM KH_2_PO_4_, 10 μM H_3_BO_3_, 0.5 mM MgSO_4_, 0.5 μM MnSO_4_, 0.2 μM CuSO_4_, 0.5 μM ZnSO_4_, 0.05 μM Na_2_MoO_4_, and 0.1 mM Fe (III)-EDTA. The pH of the solution was adjusted by adding a 1 N HCL solution to 5.5. In −Fe+Zn and +Fe−Zn treatments, the Fe (III)-EDTA and ZnSO_4_ were not added respectively in the control solution. While in −Fe−Zn treatment both Fe (III)-EDTA and ZnSO_4_ were not added in the control solution (Table 1).

**TABLE 1:** Details of the nutrient stress treatments given to the seedlings of the pearl millet inbred ICMB 1505.

S. No.	Treatment	Nutrient solution details	Stress level
1	Control (+Fe+Zn)	Presence of both Fe (III)-EDTA and ZnSO4	No stress
2	Treatment (+Fe−Zn)	Presence of Fe (III)-EDTA and the absence of ZnSO4	Zn stress
3	Treatment (−Fe+Zn)	Absence of Fe (III)-EDTA and presence of ZnSO4	Fe stress
4	Treatment (−Fe−Zn)	Absence of both Fe (III)-EDTA and ZnSO4	Fe and Zn stress

Customized 96-well PCR plates with holes at the bottom side were used for supporting the seedlings for the effective harvesting of roots and leaves after the treatment period. After placing the germinated seedlings in the PCR plates with roots emerging out on the bottom side, the plates were left floating in the treatment solutions containing trays. Each tray was accommodated with three plates, indicating three replicates per treatment. Three to four days later, seedlings (nearly 40 per tray) were transferred to the respective treatment and were maintained for 12 days (20).

### RNA-sequencing

Total RNA was extracted from the replicated leaf and root samples of control (+Fe+Zn) and stress treatments (−Fe−Zn, −Fe+Zn, and+Fe−Zn) using QIAGEN RNeasy Plant Mini Kit. Genomic DNA contamination was removed with RNase-Free DNase (QIAGEN). The purity of RNA, i.e., RNA degradation and DNA contamination were checked by agarose gel electrophoresis. The Total RNA was quality checked using RNA 6000 Nano Kit (Agilent Technologies, USA) on 2100 Bioanalyzer (Agilent Technologies, USA), with a minimum RNA Integrity Number (RIN) value of 7. RNA concentrations were determined with a NanoDrop ND-8000 spectrophotometer (Nano-Drop Technologies; THERMO Scientific, Wilmington, DE, USA). Poly(A) messenger RNA (mRNA) was purified from the total RNA using oligo-dT attached magnetic beads, to capture for polyA tails, using two rounds of purification. The RNA was fragmented into 200–500 bp fragments during the second elution of poly-A RNA using an ultrasonicator. The Superscript-II reverse transcriptase (Life Technologies, Inc.) and random primers were used to copy the cleaved RNA fragments into first-strand cDNA. After second-strand cDNA synthesis, fragments were end-repaired and A-tailed, and indexed adapters were ligated. The purified products enriched with PCR were used to generate the final cDNA library. RNA-Seq libraries for all samples were prepared using NEBNext UltraII RNA library preparation kit for Illumina; Cat. no. E7770 (New England Biolabs), according to manufacturers recommended protocol. The tagged cDNA libraries were used for 2 × 150-bp paired-end sequencing by pooling them in equal ratios onto a single lane of the Illumina HiSeq4000. Sequencing was done by 2× 150 bp paired-end chemistry of Illumina HiSeq 4000 using generated Illumina clusters loaded onto Illumina Flow Cell.

The quality of the raw reads obtained by sequencing was checked by the FASTQC online tool. The Trimmomatic tool was used for filtering low-quality reads and adaptor sequences (22) followed by assessing the data with the FASTQC. Finally, the obtained clean reads were aligned to the pearl millet reference genome (23) using hisat2 (24) to reconstruct the transcriptome and perfectly aligned sequences were considered for further analysis. The alignments were converted to a sorted bam format using Samtools (25).

### Differential gene expression and pathway enrichment analysis

The sequence reads per each genomic feature were measured with featureCounts (26).^2^ DESeq2 R Package was used in determining the gene expression differences between control and treatments (27). The combined features for each combination of control and treatment sample data were fed to R package DESeq (27) to measure the DEGs. DEGs were selected based on fold-change (FC) and FDR-corrected *p* < 0.05 and classified as upregulated (≥+1.5 FC) or downregulated (≤−1.5 FC). The identified DEG sequences were further Blast (28) and compared with the Viridiplantae protein sequence from the UniProt database to assign the associated annotations. With the known gene ontology (GO) terms from the annotations, the Cytoscape plugin Bingo was used to make the metabolic pathway.

### Validation of RNA-sequencing data by real-time PCR

To check the accuracy of transcriptomic analysis, data validation by qRT-PCR was carried out. The 17 selected DEGs were chosen for validation (Supplementary Figure 1) and the cDNA was synthesized from the extracted RNA from the samples. Normalization of all the cDNA samples was done to equalize the concentrations of all the samples. Primers were designed for the chosen DEGs using Primer3plus software (Supplementary Table 1). To normalize the data, the actin gene (*PgActin*) of pearl millet was used as a reference gene. The ΔΔCT method was used for the calculation of the relative gene expression of targeted genes (29).

### Identification of gene orthologs

The top 68 Fe and Zn homeostasis pathway-related genes identified in pearl millet through RNA-Seq were BLAST searched against the genomes of *Arabidopsis* spp., maize, rice, sorghum, and foxtail millet to identify the orthologs. All the hits with at least 70% similarity were considered significant. The orthologous relationships between pearl millet and the other five crop species were visualized in the circos plots using the ClicO FS tool (30). The orthologs of a gene were aligned using Bioedit sequence alignment editor software version 7.2.5 for the identification of haplotypes (31).

## Results

### RNA-sequencing

Twenty-four leaf and root samples of Fe and Zn stress-induced pearl millet inbred ICMB 1505 were subjected to genome-wide transcriptome sequencing using a paired-end method with three biological replicates. A total of 841 million reads accounting for 39 GB of data was generated. After trimming the low-quality reads, a total of 753 million reads were aligned to the reference genome. Ultimately, of the 562 million high-quality successfully mapped reads, over 78.57% were observed to be uniquely mapped reads and 21.10% were multiple mapped reads (Table 2).

**TABLE 2:** Total and mapped reads obtained from different treatments of Fe and Zn stresses in leaf and root samples of pearl millet through RNA-Seq analysis.

Sample ID	Tissue	Treatment	Raw reads	Filtered reads	Alignment reads	Alignment rate (%)	Unique mapped reads	Unique mapped reads (%)	Multiple mapped reads	Multiple mapped reads (%)
T1	Leaf	+Fe+Zn	36795692	32809362	28250596	86.11	19327977	68.42	8922619	31.58
T2	Root	+Fe+Zn	33824036	30018012	26174715	87.2	20404340	77.95	5778409	22.08
T3	Leaf	+Fe+Zn	41783596	37190222	31715264	85.28	24260737	76.5	4370771	13.78
T4	Root	+Fe+Zn	33948826	30712570	25190307	82.02	17980023	71.38	3155284	12.53
T5	Leaf	+Fe+Zn	40559716	35966150	31266122	86.93	20995588	67.15	2463312	7.88
T6	Root	+Fe+Zn	33906822	31641708	17895047	56.56	13224359	73.9	2949473	16.48
T7	Leaf	+Fe−Zn	34095324	31130340	24504705	78.72	18437836	75.24	3367225	13.74
T8	Root	+Fe−Zn	22642636	19698618	16691728	84.74	12241161	73.34	2066750	12.38
T9	Leaf	+Fe−Zn	39145306	35702360	23984228	67.18	18646736	77.75	4559974	19.01
T10	Root	+Fe−Zn	33953090	30666148	18742678	61.12	16972046	90.55	8567585	45.71
T11	Leaf	+Fe−Zn	37231716	31305440	23893854	76.32	17922925	75.01	2553549	10.69
T12	Root	+Fe−Zn	33841022	31614084	23138671	73.19	16478780	71.22	2517123	10.88
T13	Leaf	−Fe+Zn	33404644	29522426	23177557	78.51	19867827	85.72	6307947	27.22
T14	Root	−Fe+Zn	33961006	31053560	20648235	66.49	16650136	80.64	5594728	27.1
T15	Leaf	−Fe+Zn	33948012	31072284	19925667	64.13	16928586	84.96	5555340	27.88
T16	Root	−Fe+Zn	33301490	29389602	22936316	78.04	19842220	86.51	7438655	32.43
T17	Leaf	−Fe+Zn	33545702	31478028	25591969	81.3	24330975	95.07	5149769	20.12
T18	Root	−Fe+Zn	33873046	31054986	13156922	42.37	11230946	85.36	3915141	29.76
T19	Leaf	−Fe−Zn	33873386	30979330	20625017	66.58	16974699	82.3	5071939	24.59
T20	Root	−Fe−Zn	33365282	29965330	25839367	86.23	20623662	79.81	6554304	25.37
T21	Leaf	−Fe−Zn	36237904	30243802	10899044	36.04	9526648	87.41	3628364	33.29
T22	Root	−Fe−Zn	33656568	29609644	22437717	75.78	17337974	77.27	3499404	15.6
T23	Leaf	−Fe−Zn	33199966	30775425	29449912	95.69	26748778	86.92	10222785	33.22
T24	Root	−Fe−Zn	46935698	40922628	35931492	87.8	24712599	68.78	4391710	12.22

### Identification of differentially expressed genes

**FIGURE 1:**
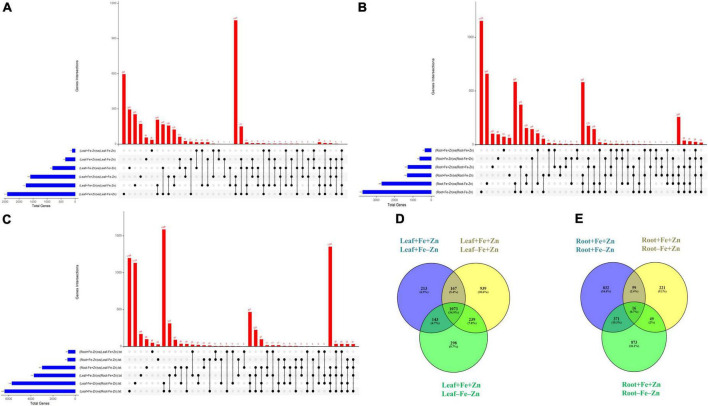
Upset plot illustrating the intersections between the set of DEGs identified in response to –Fe, –Zn, –Fe–Zn stresses in **(A)** leaf, **(B)** root, and **(C)** leaf and root. The blue bar on the left side indicates the total number of DEGs expressed under specific stress treatments and the red bar on the right side indicates the DEGs unique to a specific treatment and between the treatments. Dots connect by a black line on the bottom panel indicate the intersections between the treatments and the representation of stress-specific and common DEGs in response to –Fe, –Zn, –Fe –Zn stresses in **(D)** leaf and **(E)** root.

A total of 37,093 DEGs were identified in control vs. stress conditions of leaf, root, and leaf-root comparisons after adjusting the *p*-value (<0.05) and log_2_ fold change value (>1.5-upregulated, <−1.5-downregulated). In leaf comparisons, more DEGs (2,418) were identified in the Fe-deficient condition and few DEGs (105) were observed in both nutrient-deficient conditions (−Fe−Zn) when compared to the Zn-deficient leaf (Figure 1A). In root comparisons, a high number of DEGs (3,737) was differentially expressed in −Fe−Zn treatment when compared to −Fe treatment whereas a low number (345) was observed in Fe-deficient root (Figure 1B). In leaf-root comparisons, the high number of DEGs (6,366) was recorded in −Fe−Zn root treatment over −Fe leaf, and a low number of DEGs (629) were found in −Fe leaf compared to −Zn root treatment (Figure 1C). The 10,194 unique DEGs identified from all comparisons were further analyzed for gene ontology studies, of which 9,981 DEGs were annotated and the remaining were unannotated besides showing a higher level of expression under stress conditions (Supplementary Figure 2).

### Gene ontology and pathway enrichment

The GO terms were assigned to the identified DEGs, of which 8,605 sequences were annotated to cellular, biological, and molecular functions. A total of 5,352 genes were annotated for 406 cellular components, 6,764 genes for 1,223 molecular functions, and 4,327 genes for the 1,475 biological process (Supplementary Figures 3A,B). Besides GO terms, KEGG (Kyoto Encyclopedia of Genes and Genomes) was used to assign biological pathways to the identified DEGs. A total of 458 DEGs were assigned to 39 pathways (Supplementary Figure 3C), of which carotenoid biosynthesis, hormone biosynthesis, isoprenoid biosynthesis, and porphyrin-containing compound metabolism pathways were root-specific. The DEGs expressed in the leaf treatments were identified to be involved in 22 pathways, of which the tRNA modification pathway was specific to leaf tissue.

### Differentially expressed genes in leaf

A total of 2,418 DEGs were expressed in Fe-deficient conditions, 1,596 DEGs in −Zn, and 1,753 DEGs under combined nutrient stress (−Fe−Zn) when compared to control (+Fe+Zn). More than 1,000 genes were commonly expressed across all stress treatments (−Fe, −Zn, and −Fe−Zn) wherein 213, 939, and 298 DEGs were unique to −Zn, −Fe, and −Fe−Zn treatments, respectively (Figure 1D). Among the genes involved in Fe and Zn uptake, *O-methyltransferases* were >7 times upregulated in all the leaf treatments (−Fe, −Zn, −Fe−Zn) when compared to control (+Fe+Zn). *Heavy metal ATPase* (HMA), *plasma membrane ATPase*, *putative ABC transporter B family member*, *ABC transporter G family member*, *calcium-transporting ATPase*, H^+^-ATPase domain-containing members gene transcripts related to metal ion transport were induced several folds, wherein, *magnesium transporter* was downregulated (1.75-fold) in all the leaf treatments. Fe storage protein, *ferritin*, and *solute protein family 40* were nearly twofold downregulated in −Fe and −Fe−Zn leaf. *Zinc-induced facilitator* (ZIF) like protein and a gene involved in Fe ion homeostasis were >1.78 times induced in −Fe condition while the *potassium transporter* gene was 2.22 times upregulated under −Fe condition. *Zinc transporter 5* was highly downregulated in −Fe leaf (−2.67) when compared to −Fe−Zn (−1.61) whereas *vacuolar cation/proton exchanger* was highly upregulated in −Fe−Zn leaf (2.15) when compared to −Fe leaf (1.89). *Mugineic-acid 3-dioxygenase* (IDS2) gene was fourfold upregulated in the presence of Fe and was 1.68-fold downregulated in the absence of Fe in the leaf. The protein involved in the Zn transmembrane transporter activity was 2.88 times upregulated in the presence of Zn (Supplementary Table 2).

The enzymes, *sucrose synthase* (twofold) and *starch synthases* (fivefold) involved in the carbohydrate biosynthesis process and alcohol dehydrogenase enzyme (>3.60-fold) were upregulated in all the leaf treatments. *Photosystem II CP47 reaction center* and *ATP synthase* were >3-fold upregulated under Fe deficiency (−Fe, −Fe−Zn). *RUBISCO*, an enzyme involved in photosynthetic processes was twofold upregulated, wherein *carbonic anhydrase* (−1.89) was downregulated in Fe-deficient condition (Supplementary Table 3). The glycolysis-associated enzymes, *glyceraldehyde-3-phosphate dehydrogenase* (>2.45), *phosphoglycerate mutase* (>4.44), *pyrophosphate-fructose 6-phosphate* (>2.35), ATP-dependent *6-phosphofructokinase* (>3.37), were upregulated in all the leaf treatments (−Fe, −Zn, −Fe−Zn). Additionally, *succinate dehydrogenase* (2.83), *phosphoenolpyruvate carboxylase* (PEPC 2.07) enzymes involved in the TCA cycle, and photorespiration process was induced in −Fe condition (Supplementary Table 4).

**FIGURE 2:**
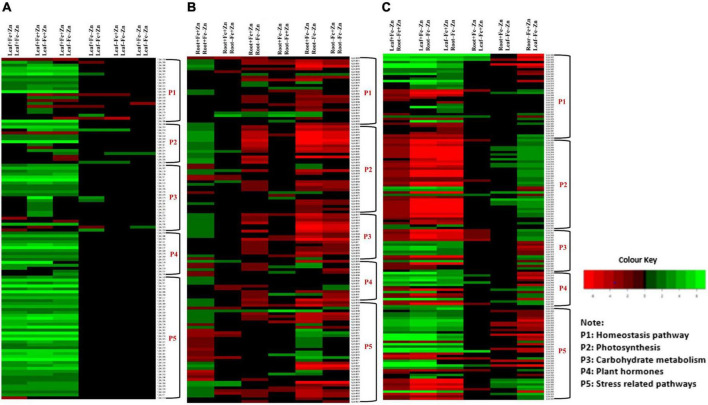
Heatmap of the selected differentially expressed genes operating under Fe and Zn stress **(A)** across leaf-pairwise combinations, **(B)** across root-pairwise combinations, **(C)** between leaf and root combinations.

Among the enzymes involved in phytohormones biosynthesis and signaling pathways, *indole-3-glycerol-phosphate synthase* (>1.66), *1-aminocyclopropane-1-carboxylate* (ACC) *synthase* (>2.81), AP2-like ethylene-responsive transcription (>4.13), *and auxin response factor* (ARF >2.15) were highly upregulated in all the stress treatments (−Fe, −Zn, −Fe−Zn). The upregulation of the auxin-responsive protein (ARP >7.45) was observed in −Fe and −Zn treatments. In all Zn-deficient (−Zn, −Fe−Zn) conditions, auxin-responsive factor 23 was upregulated by 2.58-fold (Supplementary Table 5). *Xyloglucan endotransglucosylase/hydrolase* (two to sevenfold), *cellulose synthase* (>7.52), and *expansin* (two to fourfold) which are involved in cell wall organization were upregulated in all stress treatments when compared to the control. Moreover, the induced expression of *S-acyltransferases* (four to sixfold), *diacylglycerol O-acyltransferases* (>4.80), and *diacylglycerol kinases* (2.41–3.64-folds) involved in the lipid metabolism were noticed across stress treatments. Further, in our study peroxidases (three to eightfold), mitogen-activated protein kinase (>1.66), NBS-LRR domain-containing proteins (>1.87), and serine/threonine kinases (>2.25) related to biotic and abiotic stress tolerance were upregulated under nutrient-starved leaf treatments. Superoxide dismutase (SOD) involved in scavenging reactive oxygen species (ROS) was threefold downregulated in Zn-deficient leaf (Figure 2A) (Supplementary Table 6).

### Differentially expressed genes in root

Iron-deficiency in root recorded 345 DEGs, Zn-deficiency showed 1,278 DEGs, and −Fe−Zn condition recorded 1,309 DEGs when compared to the control. A total of 16 genes were commonly regulated in all the stress treatments (−Fe, −Zn, −Fe−Zn) while 833, 221, and 873 DEGs were unique to −Zn, −Fe, and −Fe−Zn treatments, respectively (Figure 1E). The downregulation of metal ion transmembrane transporter activity genes was observed in all the stress treatments (−Fe, −Zn, −Fe−Zn) when compared to the control. In Fe-deficient conditions, *ferritin* protein, 2-oxoglutarate (2OG), HMA, *solute carrier family 40 protein*, *potassium transporter*, and *copper transporter* were downregulated by several folds. In −Fe root, the induced expression of *3″ deamino-3″ oxonicotianamine reductase* (2.27), *nicotianamine synthases* (NAS 2.59), *adenine phosphoribosyltransferase* (APRT >2.03 times) genes, and in −Zn root, the upregulation of *ribose 5-phosphate isomerase* (1.74) gene involved in phytosiderophore (PS) synthesis were observed. The expression of MYB transcription factor was 2.45-fold induced in −Zn root and in −Fe−Zn condition it was 2.8-fold downregulated. The gene involved in the Zn transport was nearly two to threefold downregulated in the −Zn, −Fe, and −Fe−Zn treatments. *Aspartate aminotransferases* (IDI4) involved in the methionine (Met) cycle were 2.39 times downregulated in −Fe−Zn when compared to the −Zn treatment. The *oligopeptide transporter 3* (OPT3) gene was 1.64 times induced in the roots under the deficiency of Fe and Zn (Supplementary Table 7).

The *magnesium chelatase*, *Mg-protoporphyrin IX chelatase*, and *chlorophyll-binding a-b proteins* involved in the chlorophyll biosynthesis pathway were several folds downregulated in the absence of Fe and were upregulated in the presence of Fe. The upregulation of light reaction involved proteins, *ferredoxin* (2.36), *ferredoxin NADP reductase* (>1.69), *ATP-synthase complex assembly* (2.42), and downregulation of *PSII reaction center* (−1.63), *cytochrome c oxidase* subunit (−1.52) and *cytochrome c* (−2.02) were observed in Zn-deficient root. *RUBISCO*, an enzyme involved in the dark reaction is nearly two times upregulated in either of the nutrient deficiency (−Fe or −Zn) but 2.62-fold downregulated under −Fe−Zn treatment (Supplementary Table 8).

Genes such as *3-Phosphoglycerate dehydrogenase* (2.45), *phosphoglycerate kinase* (2.07), *malate dehydrogenase* (1.54), *fructose-bisphosphate aldolase* (2.09), *glyceraldehyde-3-phosphate dehydrogenase* (2.41), PEPC (2.21), *phosphoenolpyruvate carboxykinase* (2.33), and *glucose-6-phosphate 1-dehydrogenase* (2.11) involved in the carbohydrate metabolism process were upregulated and *lactate dehydrogenase* (−1.68) was downregulated in the Zn-deficient root. The downregulation of *pyruvate* (−4.53), *3-phosphoglycerate dehydrogenase* (−2.84), *phosphoglycerate kinase* (−1.64), *malate dehydrogenase* (−1.51), *fructose-bisphosphate aldolase* (−3.04), *phosphoenolpyruvate carboxykinase* (−3.68), *phosphoglycerate mutase* (−1.72), *isocitrate lyase* (−1.81), *pyruvate dehydrogenase E1 component* (−1.99), *aconitase hydratase* (−1.64), and *D-fructose-1,6-bisphosphate 1-phosphohydrolase* (−2.45) was observed in −Fe−Zn condition (Supplementary Table 9).

In the case of phytohormones, the downregulation of *auxin efflux carrier protein* (−1.59) and ARF (−2.15) was observed in Fe-deficient roots. In the −Zn root, the downregulation of *tryptophan synthase* (−1.52), ARP (−2.23) was recorded. The expression of ARF SAUR36 was induced two to threefold when the root was deficient to −Fe or −Zn. The downregulation of ACC *synthase* (−1.87) was noticed in the −Fe−Zn treatment (Supplementary Table 10). The downregulation of *trehalose 6-phosphate phosphatase* (<−1.94) involved in cell wall organization was observed in −Fe and −Zn root treatments, while downregulation of *cellulose synthase* (−1.52), *xyloglucan endotransglucosylase/hydrolase* (<−1.71) were observed in −Fe−Zn root. In all the root treatments, the *expansin* was nearly twofold upregulated. *Diacylglycerol O-acyltransferases* (−2.21) involved in the lipid metabolism were downregulated in −Fe−Zn condition whereas *S-acyltransferases* (−1.68) were downregulated in Fe-deficient roots. Peroxidases (<−1.7) were downregulated in all the root treatments. Serine/threonine kinases (−2.65) involved in the stress signaling mechanism were downregulated in Fe-deficient conditions (Figure 2B) (Supplementary Table 11).

### Tissue-specific expression of genes

Leaf and root stress treatments were compared to identify the tissue-specific expression of genes involved in uptake and transport activities. The upregulation of PS synthesis enzymes, *formate dehydrogenases* (FDH > 6.73), *3″ deamino 3″ oxonicotianamine reductase* (>4.46), NAS (five to ninefold), involved in the uptake of Fe and Zn from the rhizosphere and ABC transporter G protein (>1.77) was observed in −Fe and −Fe−Zn root when compared to −Fe and −Zn leaf. FER-like transcription factors, *2′-deoxymugineic-acid 2′-dioxygenase* (IDS3 four to eightfold), heavy metal detoxification proteins (>1.76), bZIP domain-containing proteins (five to sevenfold), calcium ATPase (>2.38) were upregulated while MYB transcription factors (<−5.39), IDS2 (<−3.72) was downregulated in −Fe and −Fe−Zn root when compared to −Fe and −Zn leaf. Downregulation of zinc transporter 5 (−2.59), and zinc transporter 3 (−1.57) was recorded in −Fe root when compared to −Zn leaf. The downregulation of ZIF-like protein (−2.84) and upregulation of *S-adenosyl methionine* (SAM) *synthase* (1.95) and 1, 2-dihydroxy-3-keto-5-methylthiopentene (3.33), 5-methyltetrahydropteroyltriglutamate (homocysteine transferases >1.5) were observed in −Fe−Zn root when compared to −Fe leaf. The gene involved in iron-nicotianamine transmembrane transporter activity was downregulated (−1.79) in −Fe root when compared to −Zn leaf. The APRT (>1.96 times), involved in the ATP production needed for the Met activation process was induced in the root treatment (−Fe, −Fe−Zn) when compared to leaf treatments (−Fe, −Zn). The IDI4 (< −2.38), potassium transporter gene (<−2.19), and *ribose 5-phosphate isomerase* (<−1.60) were downregulated in the root treatment (−Fe, −Fe−Zn) but the same genes were upregulated in −Fe−Zn leaf (Supplementary Table 12).

The expression of *delta-aminolevulinic acid dehydratase* (<−1.54) involved in chlorophyll biosynthesis was low in the −Fe, −Fe−Zn root treatments when compared to −Fe and −Zn leaf. The *RUBISCO* (>1.54), an important plant sugar synthesis enzyme of dark reaction was upregulated in −Fe−Zn leaf when compared to root deficient in −Fe or −Zn but downregulated in root −Fe (<−2.5) and −Fe−Zn (<−6.8) when compared to Zn-deficient leaf (Supplementary Table 13). The enzymes, *pyruvate* (two to sevenfold), *3-Phosphoglycerate dehydrogenase* (two to fivefold), *malate dehydrogenase* (two to fivefold), and *phosphoglycerate kinase* (three to fivefold) involved in the carbohydrate metabolism were downregulated in the root treatments (−Fe, −Fe−Zn) when compared to leaf treatments (−Fe, −Zn). The above-mentioned genes were upregulated in −Fe−Zn leaf when compared to −Fe root and downregulated in −Fe leaf when compared to −Zn root. The *glyceraldehyde-3-phosphate dehydrogenase* (−2.72), *pyruvate kinase* (−1.70), PEPC (−2.20), and *pyrophosphate fructose 6-phosphate* (−2.04) were downregulated in −Fe−Zn leaf when compared to −Fe or −Zn leaf (Supplementary Table 14). In the case of genes involved in phytohormones synthesis and signaling mechanism, ARP (>3.23), auxin efflux carrier component (three to ninefold), ACC *synthase* (two to threefold), were upregulated and, ARP SAUR36 was two to sixfold downregulated in −Fe and −Fe−Zn root when compared to −Zn and −Fe leaf tissue (Supplementary Table 15).

Serine and threonine kinases were nearly 2–5 times upregulated in −Fe, −Fe−Zn root when compared to −Fe and −Zn leaf treatments. *Patatin, laccase, kinesin*, SOD, were two to eightfold upregulated and, *serine/threonine-protein phosphatase, catalase*, and *ascorbate peroxidases* were two to ninefold upregulated in root treatments (−Fe, −Fe−Zn) when compared to leaf treatments (−Fe, −Zn) and four to fivefold downregulated in −Fe−Zn leaf when compared to −Fe root. *Trehalose 6-phosphate phosphatase* (<−3.75), and *peroxidases* (<−3.67) were downregulated in −Fe−Zn leaf when compared to −Fe and −Zn root (Figure 2C) (Supplementary Table 16).

### Chromosomal annotation of identified iron and zinc responsive genes

**FIGURE 3:**
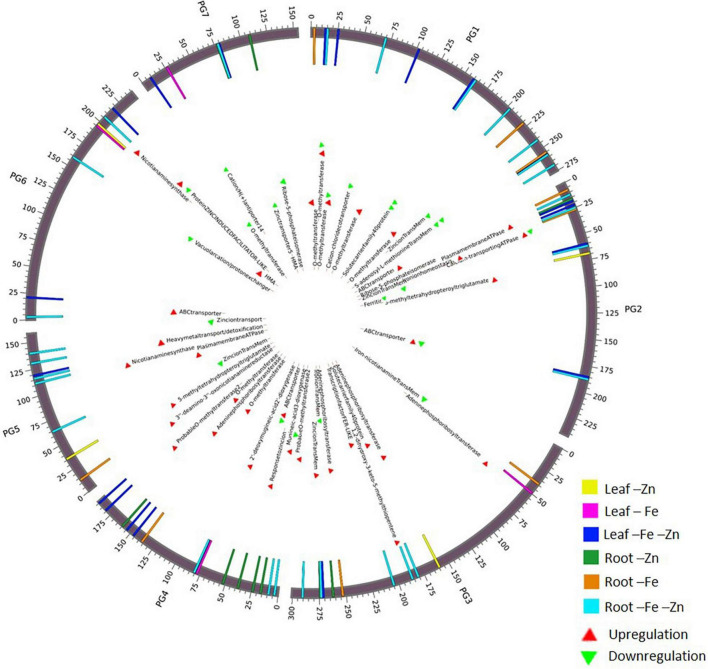
Circos plot representing important Fe and Zn homeostasis-related genes along with the differential expression patterns in pearl millet.

A total of 68 genes (Supplementary Table 17) corresponding to Fe and Zn uptake and transport, identified in our study were distributed on all seven chromosomes (Figure 3). The genes related to Fe response and transport were distributed on 2 and 3 chromosomes while for Zn, they were distributed on all the chromosomes except on chromosome 2. The genes of the MA pathway for PS synthesis were distributed on all 7 chromosomes. OPT3 and ferritin were located on chromosome 2 and the transcription factor FER-like was located on chromosome 3.

### Identification of orthologs

The identified 68 Fe- and Zn-homeostasis pathway-related genes (Supplementary Table 17) in the present study were BLAST searched against *Arabidopsis* spp., rice, maize, sorghum, and foxtail millet genomes for the identification of orthologous genes.

**FIGURE 4:**
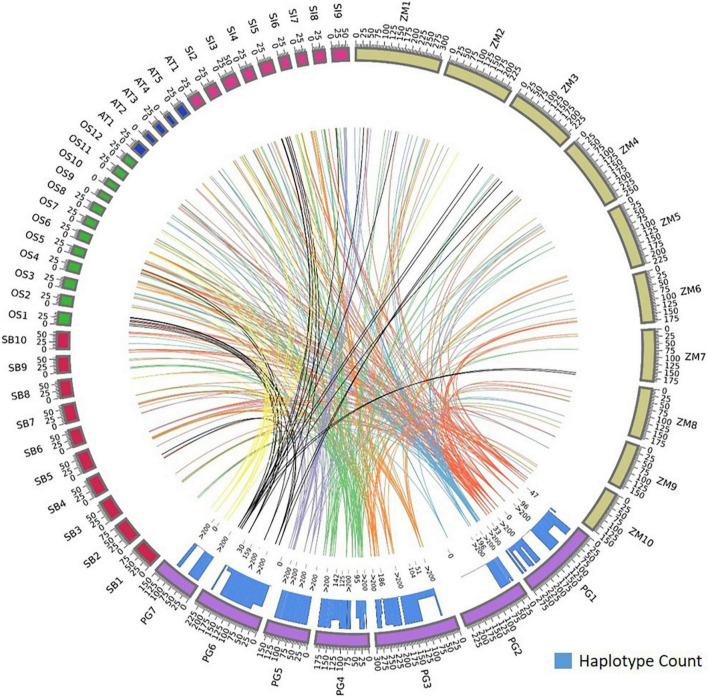
Comparative map showing the orthologous genes of Fe and Zn homeostasis in pearl millet to rice, maize, sorghum, foxtail millet, and *Arabidopsis* spp. and the histogram within circos plot represents the haplotypes count of identified orthologous genes when aligned to pearl millet gene sequences.

Foxtail millet had the maximum homology, sharing 64 orthologs with pearl millet followed by rice (32), maize (33), sorghum (34), and *Arabidopsis* spp. (8). Annotations were assigned to the identified orthologs to know their functionality in respective species. The foxtail millet had the highest similarity to the pearl millet genes by capturing 80 ortholog sequences from 64 genes with 23 sequences of 100% similarity. In rice, 92 ortholog sequences were identified for 58 genes, of which 11 sequences showed 100% similarity and 34 sequences showed >90% similarity to pearl millet genes. Maize had 102 ortholog sequences for the 56 pearl millet genes, of which 11 sequences recorded 100% similarity. The pearl millet to sorghum comparison yielded 93 ortholog sequences from 32 genes, of which 16 sequences showed 100% similarity. In *Arabidopsis* spp. a total of 14 ortholog sequences were identified for 8 genes, of which 10 ortholog sequences recorded >90% similarity (Figure 4).

Our analysis also showed more than one ortholog for a given gene. Among food crops, 23 genes out of total identified 58 orthologs in rice reported more than one ortholog sequence. Maize, sorghum, and foxtail millet had 32, 23, and 11 genes, respectively, and had more than one ortholog.

The orthologs of four genes, *Ribose 5-phosphate isomerase*, SAM synthase, FDH, and OPT3 were identified in Arabidopsis and four food crops. The ortholog of a gene, 5-methyltetrahydropteroyltriglutamate—homocysteine was identified in Arabidopsis and three food crops except for sorghum, and the ortholog of one gene, *ABC transporter F* family member was identified in Arabidopsis and three food crops except for foxtail millet. The *plasma membrane ATPases* orthologs were identified in *Arabidopsis* spp. and three food crops except for rice. More than 40 gene orthologs were identified in all food crops except *Arabidopsis* spp. The orthologs of eight genes were identified in three food crops except for rice and the orthologs of two genes were identified in three food crops except for sorghum.

### Identification of haplotypes

**FIGURE 5:**
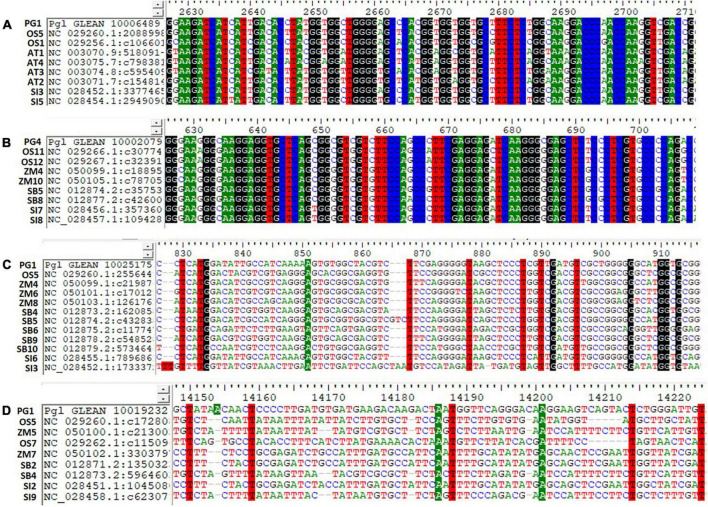
Haplotypes representation for the top identified Fe and Zn genes across Arabidopsis and five food crops (AT, *Arabidopsis thaliana*; PG, *Pennisetum glaucum*; OS, *Oryza sativa*; ZM, *Zea mays*; SB, *Sorghum bicolor*; and SI, *Setaria italica*). **(A)** Pgl_GLEAN_10006489 (SAM synthase) with ortholog sequences of *Arabidopsis* spp. and two food crops showing highly conserved regions of a gene, **(B)** Pgl_GLEAN_10002079 (Ferritin) with ortholog sequences of four food crops showing highly conserved regions of a gene, **(C)** Pgl_GLEAN_10025175 (O-methyltransferase activity) with ortholog sequences of four food crops showing higher variability regions with a few haplotypes of a gene, and **(D)** Pgl_GLEAN_10019232 (SAM transmembrane transporter activity) with ortholog sequences of four food crops showing higher variability regions with a few haplotypes of a gene.

The identified ortholog sequences of 68 pearl millet Fe and Zn-related genes across *Arabidopsis* spp. and four food crops were fetched out and aligned for the identification of haplotypes. The ortholog sequences of all four pearl millet genes (*Ribose-5-phosphate isomerase*, SAM synthase, FDH, and OPT3) identified across the *Arabidopsis* spp. and four food crops (rice, maize, sorghum, and foxtail millet) recorded >200 haplotypes (Figure 4). A total of >200 haplotypes were identified in the ortholog sequences of four pearl millet genes-*Plasma membrane ATPases* (except rice), *ABC transporter F family member 1* (except foxtail millet), and two 5-methyltetrahydropteroyltriglutamate—homocysteine (except sorghum), identified across *Arabidopsis* spp. and any of the three food crops. The maximum haplotypes (>200) were recorded in the ortholog sequences of 29 pearl millet genes with all food crops. The minimum haplotypes (35) were identified in the ortholog sequences of the SAM transmembrane transporter activity gene across four food crops when aligned to pearl millet genes (Figure 5).

## Discussion

The present study was performed to determine the expression pattern of Fe and Zn-responsive genes in leaf and root tissues under starvation conditions. The expression changes of genes involved in different cellular pathways that consequently effecting the Fe and Zn uptake and transport mechanism have been investigated.

### Altered expression of cellular pathways and homeostasis genes under iron deficiency

**FIGURE 6:**
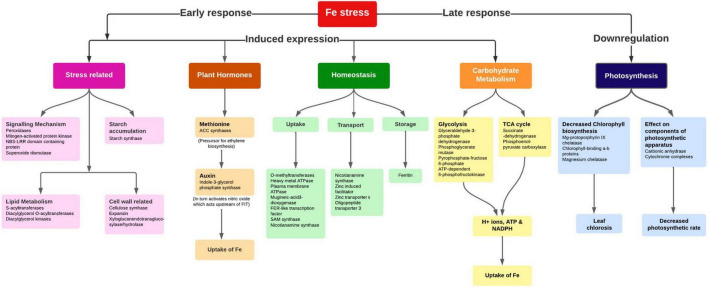
Important pathways and their interconnectivity in response to Fe stress in pearl millet.

Plants uptake Fe from the soil by reduction-based strategy at the root surface, wherein expression of metal ion uptake genes FRO2 (ferric chelate reductase oxidase), and IRT2 (iron regulated transporter) were upregulated in Fe starvation to avoid deficiency. The induced expression of a bHLH transcription factor named FER in tomato (34) or FIT1 (Fe-induced transcription factor 1) in Arabidopsis (35), positively controlling the Fe uptake genes was observed in the present study (Figure 6). In a reduction-based strategy, the plasma membrane’s H^+^-ATPase activity of releasing protons to the rhizosphere led to Fe-deficiency to decrease the pH of the soil which solubilizes the Fe^3+^ and drives the Fe ions by generating the electrochemical proton gradient (36). Fe in graminaceous plants is mostly taken by the roots by chelation activity (Strategy II), where plants synthesize the MA family of PS from SAM a conserved pathway (37). The enzymes, SAM synthetase, S adenosylmethionine decarboxylase, NAS, IDS3, and 3″’deamino 3″’oxonicotianamine reductase for the PS production were upregulated in the Fe-deficient root indicating their sensitivity to Fe nutrition. Moreover, IDS2, one of the MA synthesis enzymes, involved in the hydroxylation of the C-3 positions of MA, and 2′-deoxymugineic acid (DMA) were downregulated in Fe-deficient root as it was a Fe-dependent enzyme (38). Hence, for the production of MAs, sufficient quantities of Met molecules are needed, which are generated by the Met cycle. The enzymes, 5 methyltetrahydropteroyltriglutamate (homocysteine methyltransferases), IDI4, and the intermediate product, 1,2-dihydroxy-3-keto-5-methylthiopentene formed by dehydratase-enolase-phosphatase (DEP) enzyme of Met cycle were induced in Fe-deficient root. The FDH, ribose 5-phosphate isomerase, APRT, enzymes synchronously expressed with Met cycle enzymes related to the Met recycling reactions (39), which were also reported to be accumulated in Fe-deficient roots.

The enhanced extrusion of protons and FC-R (ferric chelate-reductase) activity during Fe-deficiency requires a higher rate of NAD(P)H and ATP regeneration aided by carbohydrate catabolism to necessitate the increased uptake (40) (Figure 6). The carbohydrate catabolism increases the accumulation of enzymes and intermediates of the glycolytic cycle for the production of ATP needed for the H^+^ ATPase activity, increases the H^+^ ions in the proton extrusion process, and reduces equivalents for FC-R activity (40). To maintain the glycolytic cycle, the feedback inhibition of *phosphofructokinase* (PFK) can be overcome by the utilization of PEP through PEPC activity (41). PEPC induces the production of organic acids resulting in the acidification of cytoplasm, indeed responsible for the extrusion of H^+^ ions from the root surface. In addition to the PEPC activity and organic acids production, an increase in CO_2_ fixation (42) and sucrose accumulation under Fe-deficient roots and shoots helped in maintaining the sugar concentrations (PEP) needed for PEPC activity (43).

Moreover, under Fe deficiency, the genes related to several additional metabolic pathways and cytosolic enzymatic activities showed elevated expression for the production of reducing equivalents required to support FC-R activity. In plants, for the transport of Fe molecules from root to shoot, the principal chelator’s citrate, malate, NA (nicotianamine), and MAs are required. Here induced expression of PEPC in Fe-deficiency, aids in the Fe uptake and transport by secretion of malate and other organic acids. Fe is known to be transported as Fe^3+^-chelates (citrate and MAs) in the xylem and as Fe^2+^-NA in the phloem. The induced expression of the NAS gene aids in NA production required for Fe translocation from root to shoot was well studied (44). The upregulated OPT3 in Fe-deficient root suggests its role in the redistribution between vegetative tissues and is also known to be involved in loading shoot Fe to phloem for transport and movement of Fe to developing seeds (45). This may be a critical part of understanding the proportional Fe uptake of biofortified varieties in comparison to non-biofortified varieties to compensate for available soil Fe concentration during the pearl millet growth and seed filling. As expected, Ferritin was downregulated in Fe-deficient leaf and root tissues since it’s a Fe storage protein.

During Fe stress, a reduced amount of ferredoxin fails to activate delta-aminolevulinic acid (ALA), affecting the chlorophyll biosynthesis and resulting in chlorosis (Figure 6). Moreover, in the chloroplast, the Fe stress affected the thylakoid proteins by reducing the light-harvesting complexes and overall amount of photosystem I and II, cytochrome complexes, rubisco carboxylation efficiency, and ATPase complex in plants (46). At the root level, some morphological changes such as the development of additional root hairs and transfer cells occur during Fe-deficiency (47), which is known to be regulated by ethylene and/or auxin signaling (48). Our study reported the induced expression of ACC *synthase* for ethylene biosynthesis and *Indole-3-glycerol-phosphate synthase* for auxin biosynthesis along with the ARP and efflux carrier protein in the Fe-deficient plant. Studies reported that the accumulation of sucrose acts as a signal for the induction of auxin under Fe-deficiency (49) which in turn is essential for the nitric oxide (NO) synthesis, required for the activation of Fe-deficiency response by acting upstream of FIT (50) (Figure 6).

The Fe starvation conditions in the pearl millet seedlings activated several genes namely, *NAS*, *SAM synthases*, *DMA*, *IDS3*, *IDS2*, *IDI4*, and *APRT* in the MA pathway and *FER*-like transcription factor, *H^+^ ATPases*, and *OPT3* in the Fe homeostasis mechanism regulating the uptake and transport. Additionally, the genes in cellular pathways indirectly aiding in the uptake and transport of Fe ions in pearl millet were also reported.

### Altered expression of cellular pathways and homeostasis genes under zinc deficiency

**FIGURE 7:**
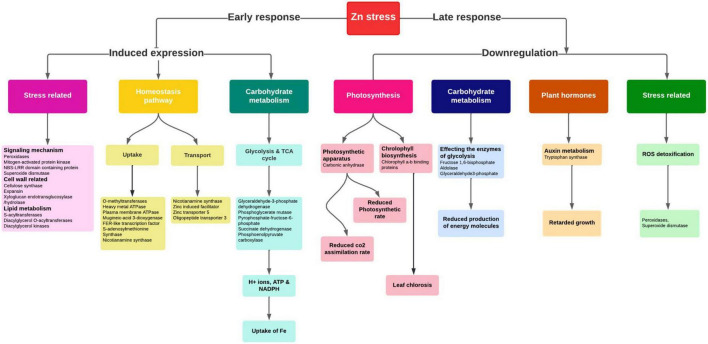
Important pathways and their interconnectivity in response to Zn stress in pearl millet.

The rhizosphere acidification process, which lowers the pH of the soil by releasing H^+^ ions and organic acids, facilitated the Zn solubilization and uptake. Plants uptake Zn in the form of Zn^2+^ and Zn^2+^-ligand complexes. The Zn^2+^-ligand complex uptake is mediated by the synthesis and release of PS, among which NA was reported to be the primary mediator responsible for the uptake and transport (51). So, under the Zn-starved conditions, the genes required for the chelator synthesis were elevated in graminaceous plants to increase the uptake from soil (Figure 7) to avoid deficiency effects. Here in our study, *SAM synthetase*, NAS, and *ribose 5-phosphate isomerase genes* for the synthesis of NA were upregulated in Zn-deficient roots similar to previous reports (20). Under Zn deficiency, the ZIP (Zinc, Iron permease family/ZRT-IRT like proteins) family transporters (Zn transporter 3 and 5) were upregulated for the uptake of remaining traces of Zn from the soil in deficiency condition (52). The members of the HMA (P-type ATPase) family for the xylem loading, maintaining the plastid Zn content were upregulated in Zn-deficient leaves (53).

Zinc acts as a co-factor in activating anhydrases, oxidases, and peroxidase enzymes and is involved in nitrogen metabolism, photosynthesis, carbohydrate metabolism, and auxin synthesis in plants (Figure 7). Zn-deficiency is known to reduce the chlorophyll content by affecting the chlorophyll biosynthesis pathway causing a decline in the photosynthetic efficiency of plants (54). Moreover, the upregulation of enzymes involved in the starch synthesis was observed which was similar to the previous report (55). The reduced expression of carbonic anhydrases was observed in Zn-deficiency affecting the entire photosynthetic rate (Figure 7). Zn-deficiency is known to affect the stomatal conductance as it is involved in maintaining the integrity of cell membrane and K^+^ uptake by which the CO_2_ assimilation rate is reduced, indirectly affecting the carbohydrate metabolism process (56). The expression of enzymes, *carbonic anhydrase, ribulose 1, 5-bisphosphate carboxylase*, involved in the photosynthesis and *fructose 1, 6-bisphosphate* involved in glycolysis are reduced in Zn starvation, as Zn is an important constituent in activating these enzymes (57). Moreover, the aldolase, sucrose, and starch synthesis enzymes are inhibited by Zn stress in plants. Under Zn deficiency, the reduced expression of SOD in leaves was observed as the enzyme needed Zn ion for the activation. The induced expression of abiotic stress-responsive peroxidases and catalase enzymes for ROS detoxification was noticed under Zn-deficiency (33). The downregulation of the tryptophan synthase enzyme involved in auxin synthesis was reported in Zn-deficiency (Figure 7), as the enzyme requires Zn ion as a co-factor, and its reduction causes disturbances in the auxin metabolism process.

Therefore, under the Zn deficiency condition, the expression of NA synthesis genes (*SAM synthetase, NAS*, and *ribose 5-phosphate isomerase* gene), ZIP family proteins, and HMA family proteins regulating the Zn homeostasis mechanism were revealed. The study also disclosed the expression changes of many cellular pathway genes which require Zn ions to activate the respective pathways.

### Cross-talks between iron and zinc pathways

The induced expression of chelator synthesis genes (e.g., NAS, DMAS, and SAM) was reported under both Fe and Zn deficiency demonstrating the dual role of PS in Fe and Zn homeostasis mechanisms in pearl millet. Several studies reported the affinity of Fe-transporters in transporting Zn along with the Fe in plants (58). Hence, the upregulation of Fe-transporters under Fe-starvation conditions resulted in increased uptake and accumulation of Zn and vice-versa. The induced expression of transcription factor FER reported under Fe and Zn deficiency aids in activating the expression of transporter genes for the uptake of both nutrients and is considered a principal transcription factor in their homeostasis pathway mechanism.

**FIGURE 8:**
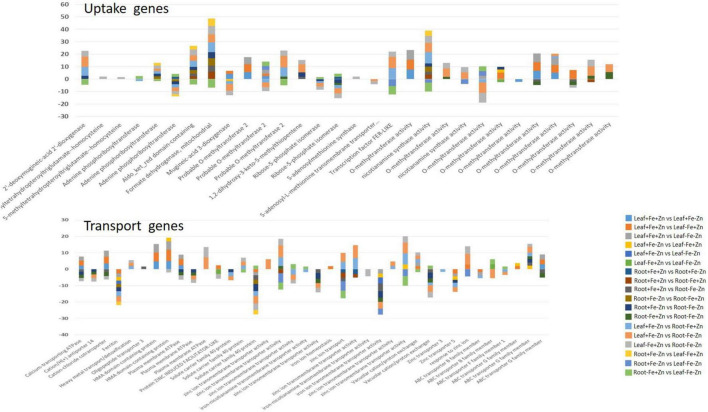
Important genes associated with the Fe and Zn uptake and transport in the leaf and root tissues of pearl millet under 18 pairwise stress combinations.

Iron-deficiency-induced accumulation of Zn ions leads to excess toxic amounts (>0.4 mg g^–1^ dry weight) of Zn in plant cytoplasm (32). Vacuoles are identified to be the main storage and detoxifying organelles for Zn excess in the plant cytoplasm. Metal tolerance protein (MTP3), HMA3, and ZIF are the vacuolar membrane transporters that aid in Zn tolerance during excess accumulation of Zn in Fe-deficient conditions. Among them, the ZIF1 transporter of the MFS (major facilitator superfamily) family has shown the induced expression in our studies which indirectly aids in the sequestration of Zn molecules by transporting chelator molecules (NA) into the vacuole (59). In addition, HMA transporters induced in Fe deficiency are probably similar to HMA3 required for the detoxification of Zn (60). This study reported the downregulated expression of zinc transporters 3 and 5 in reducing the Zn content, to avoid toxic effects due to its accumulation under the Fe deficiency condition. The same transporters can be upregulated for increasing the Zn content in target-oriented biofortification processes. Further, the studies reported that the Zn-deficiency will accumulate higher Fe by induced expression of uptake genes in plants (61). NAS involved in PS synthesis and NA production that is needed for the uptake and transport of Fe and Zn intracellularly was upregulated (44) in the current study (Figure 8).

Through an *in silico* approach, the 68 important Fe and Zn-related gene orthologs were identified in *Arabidopsis* spp. and four food crops namely rice, maize, sorghum, and foxtail millet (Figure 4). The identified ortholog sequences were grouped under pair-wise type orthologs showing a one-to-many (1: m) relationship where one gene of pearl millet has more than one ortholog in *Arabidopsis* spp. and other food crops which imply that the gene may be duplicated in an ancestor, after the event of speciation process (62). In total, the orthologous sequences of 64 genes out of 68 were identified in the foxtail millet crop with a maximum number of orthologs having 100% similarity when compared to *Arabidopsis* spp. and other three food crops explaining the fact that it had a common ancestor during the evolutionary process or belongs to the same clade in a phylogenetic tree. In the other three food crops (rice, maize, and sorghum), the orthologs for more than 32 genes were identified, when compared to *Arabidopsis* spp., which shows that all the food crops and pearl millet are related or grouped under one cluster. The ortholog sequences for the least number of genes were identified in *Arabidopsis* spp. when compared to the other four food crops, which shows that it is distantly related to the pearl millet crop.

Haplotypes are the specific group of jointly inherited DNA regions or nucleotides from a single parent (63). The genes *Ribose-5-phosphate isomerase*, SAM synthase, FDH, and OPT3 identified across the *Arabidopsis* spp. and four food crops showed highly conserved regions with a high number of haplotypes. These are the core genes strongly conserved at the nucleotide sequence level throughout the related genomes. A high number of haplotypes observed in 29 pearl millet genes with all four food crops probably indicated the conservation of these sequences during the evolution process (64). SAM transmembrane transporter activity gene showed higher variability or polymorphism by recording a few haplotypes (35) due to deletion, addition, substitution, or mutation during the process of evolution (64, 65). The continuous series of haplotypes detected for the Fe and Zn responsive genes across the *Arabidopsis* spp. and four food crops could be used for designing the common markers for its utilization across the crop species (66).

## Conclusion

A few studies in pearl millet reported a positive and significant correlation between Fe and Zn contents (15, 16), which implies the possible existence of common uptake and regulatory pathways operate to maintain genotypic variations for these two micronutrients within the limit of available soil Fe and Zn concentration during the crop growth and development thereby to seeds. Our current study revealed the expression of *NAS*, *SAM synthases*, *DMA*, *IDS3*, *IDS2*, *IDI4*, and *APRT* in the MA pathway and *FER*-like transcription factor, *H^+^ ATPases*, *HMA*, *ZIF1*, *OPT3*, and *Zn transporter 3* and *5* involved in the uptake and transport mechanism of Fe and Zn ions. The expression changes of major cellular pathway genes related to phytohormones, chlorophyll biosynthesis, photosynthesis, stress-induced pathways, carbohydrate metabolism, and their regulation in Fe and Zn homeostasis under stress conditions in pearl millet were revealed. This study is limited to the seedling stage and hence merits further investigation on the field-based study that mimics similar conditions (deficiency of soil Fe and Zn) to validate its practical application in pearl millet breeding and biofortification of varieties. Indian Council of Agricultural Research (ICAR) endorsed the minimum standards for Fe (>42 ppm) and Zn (>32 ppm) in pearl millet variety and hybrid testing and release policy since 2018 (67). Furthermore, the validated genic SNPs associated with Fe and Zn pathways will help in the development of Fe and Zn-efficient biofortified pearl millet lines through a marker-assisted selection program.

Maintaining the higher Fe and Zn with the higher yield is a challenge to the breeders using conventional breeding approaches, therefore, the present study provides the opportunity to develop and explore the genomic-assisted breeding pipelines to deliver more nutritious varieties under varied soil Fe/Zn availability in the crop ecology. Further, the above-identified genes can be used for the enhancement of Fe and Zn contents in other staple crops, which will help to ameliorate malnutrition.

## Data availability statement

The data presented in the study are deposited in the Sequence Read Archive repository, accession number PRJNA819827.

## Author contributions

CG, VS, MG, and NT conceptualized the experiment and wrote the manuscript. CG, VS, RM, AV, and HK conducted the experiments. CG, AR, JS, SR, and NT performed the data analysis. All authors read and approved the manuscript.

## Footnotes

1
http://www.wmo.int

2
http://subread.sourceforge.net
