# Supplementary material for: Identification of iron and zinc responsive genes in pearl millet using genome-wide RNA-sequencing approach
Source: Front Nutr. 2022 Nov 9;9:884381. doi: 10.3389/fnut.2022.884381 (PMC9682069; doi:10.3389/fnut.2022.884381)
Supplement: Supplementary file 1 [file Data_Sheet_1.docx]

**Identification of iron and zinc responsive genes in pearl millet through genome-wide RNA-Seq approach**

Chengeshpur Anjali Goud^1^, Vanisri Satturu^1^, Renuka Malipatil^2^, Aswini Viswanath^2^, Janani Semalaiyappan^2^, Himabindu Kudapa^3^, Santosha Rathod^4^, Abhishek Rathore^3^, Mahalingam Govindaraj^3,5^, Nepolean Thirunavukkarasu^2^

**Table S1. Primer detail of genes identified in various pathways under Fe and Zn treatments through RNASeq for qRT-PCR validation**

|  |  |  |  |  |  |  |  |  |
| --- | --- | --- | --- | --- | --- | --- | --- | --- |
| **Gene IDs** | **Gene ontology** | **Type** | **Sequence** | **Start** | **Length** | **Tm** | **GC %** | **Amplicon size** |
| Pgl_GLEAN_10001420 | Zinc ion transport | Forward Reverse | TCGTCATCCTGTTCGCATTC TCGTCCTCGTCGTCCTTATT | 214 535 | 20 20 | 62.336 62.457 | 50 50 | 322 |
| Pgl_GLEAN_10002079 | Intracellular sequestering of iron ion  iron ion transport | Forward Reverse | GTCGAGTACAATGCCTCCTATG TGCTCAATCTGCTGGGTATG | 629 1169 | 22 20 | 62.009 61.999 | 50 50 | 541 |
| Pgl_GLEAN_10002443 | ubiquinol-cytochrome-c reductase activity  Mitochondrial electron transport | Forward Reverse | ATACGAGGCTGTGCCTATTTC CTGGGCGTAAGTGCCATATAA | 535 897 | 21 21 | 61.995 62.028 | 47.6 47.6 | 363 |
| Pgl_GLEAN_10005019 | Iron-nicotianamine transmembrane transporter activity Response to iron ion | Forward Reverse | GCCACGCCGAGTCTTTATTA GGCTCTAAGTTCTCCTGTTGTT | 493 935 | 20 22 | 62.111 62.102 | 50 45.5 | 443 |
| Pgl_GLEAN_10007471 | Carbonate dehydratase activity | Forward Reverse | GGTCTGGTTCGCCATATCTATAA GTGAAGGTGGGTGGGATAAA | 956 1213 | 23 20 | 61.636 61.675 | 50 50 | 258 |
| Pgl_GLEAN_10008622 | O-methyltransferase activity | Forward Reverse | CCCACGCATTCACTACTCTTAG TATCGTTGAAGGCACCACATAG | 552 966 | 22 22 | 62.187 62.187 | 50 45.4 | 415 |
| Pgl_GLEAN_10012139 | Dioxygenase activity | Forward Reverse | GCGCATGATCCTTCCCTAATA GTAGTCGCAGTAGTTCCATAGC | 215 702 | 21 22 | 61.864 61.926 | 47.6 50 | 488 |
| Pgl_GLEAN_10012759 | Zinc ion transmembrane transporter activity | Forward Reverse | CTCGTCCCAGTCAGTTTCTTT CCATCAGTCTCCCTCACAAATAG | 1134 1472 | 21 23 | 61.995 62.08 | 47.2 47.8 | 339 |
| Pgl_GLEAN_10015686 | Nicotianamine synthase activity | Forward Reverse | GCTGCCACTAACAACAGAAAC CTTTCCCTACTACCGCAACTAC | 13 780 | 21 22 | 61.842 61.915 | 47.6 50 | 768 |
| Pgl_GLEAN_10017887 | Magnesium chelatase activity  Chlorophyll biosynthetic process Photosynthesis | Forward Reverse | GTCTGCACCATAACCAGAGTAG TAGGGTCCAGTAAGGAGGTTAG | 1262 1558 | 22 22 | 61.96 61.959 | 50 50 | 297 |
| Pgl_GLEAN_10018408 | DNA-binding transcription factor activity | Forward Reverse | CCAGTTCGTTGCTGGTAGAA CATGGGACAGGTGCTTAGTT | 2123 2763 | 20 20 | 62.005 61.922 | 50 50 | 641 |
| Pgl_GLEAN_10020996 | 2'-deoxymugineic-acid 3-dioxygenase activity  Mugineic-acid 3-dioxygenase activity | Forward Reverse | GCTGATGACCCTGATGAAGAA AGACGAGAGCAAGCCAAATAG | 589 927 | 21 21 | 61.961 62.143 | 47.6 47.6 | 339 |
| Pgl_GLEAN_10025753 | Transmembrane transporter activity | Forward Reverse | CCCAGTATACCACAGAGCAATC TAGCCCTGCTGCCTAAATAAC | 3023 3864 | 22 21 | 62.091 62.052 | 50 47.6 | 842 |
| Pgl_GLEAN_10026643 | Zinc ion transmembrane transporter activity | Forward Reverse | ACCTACCAACCAACCAATCC GCCATCCATCACACTGAACTA | 437 959 | 20 21 | 61.96 61.935 | 50 47.6 | 523 |
| Pgl_GLEAN_10030145 | Nicotianamine synthase activity | Forward Reverse | GTAGCGAACCAGGAGATCATAC CTTCCCGTACTACAGCAACTAC | 1461 2358 | 22 22 | 61.764 61.852 | 50 50 | 898 |
| Pgl_GLEAN_10033886 | Protein dimerization activity | Forward Reverse | CAAAGGTGGTGGAGTAGGTATTT TTCTTGACCCAGGCATTGTAG | 522 1086 | 23 21 | 62.245 62.193 | 43.5 47.6 | 565 |
| Pgl_GLEAN_10036448 | Zinc ion transmembrane transporter activity | Forward Reverse | CCGAGTACTTCTCCTCTGTAGT TGGTTAGGCTCAGCTCATTTC | 120 622 | 22 21 | 62.029 62.202 | 50 47.6 | 503 |

| **Table S2: List of DEGs related to Fe, Zn uptake and transport identified in response to –Fe, –Zn, –Fe–Zn stress in leaf tissue** | | | | | | | |
| --- | --- | --- | --- | --- | --- | --- | --- |
|  |  |  |  |  |  |  |  |
| **Description** | **Gene IDs** | **Leaf+Fe+Zn Leaf+Fe–Zn** | **Leaf+Fe+Zn Leaf–Fe+Zn** | **Leaf+Fe+Zn Leaf–Fe–Zn** | **Leaf+Fe–Zn Leaf–Fe+Zn** | **Leaf+Fe–Zn Leaf–Fe–Zn** | **Leaf–Fe+Zn Leaf–Fe–Zn** |
| Magnesium transporter OS=Setaria | Pgl_GLEAN_10037628 | -1.75 | -1.78 | -2.55 |  |  |  |
| Mugineic-acid 3-dioxygenase OS=Hordeum vulgare | Pgl_GLEAN_10020996 | 4.15 | 2.46 |  | -1.63 |  |  |
| Plasma membrane ATPase OS=Setaria | Pgl_GLEAN_10008446 | 2.44 | 3.81 | 2.64 |  |  |  |
| HMA domain-containing protein | Pgl_GLEAN_10030576 | 4.70 | 5.42 | 5.18 |  |  |  |
|  | Pgl_GLEAN_10000945 | 3.65 | 4.03 | 4.09 |  |  |  |
|  | Pgl_GLEAN_10006197 | 4.91 | 6.92 | 5.19 | 2.11 |  |  |
| Putative ABC transporter B family member | Pgl_GLEAN_10037353 | 1.80 | 1.95 | 1.81 |  |  |  |
| ABC transporter G family member | Pgl_GLEAN_10023174 | 2.96 | 2.89 | 3.21 |  |  |  |
| Calcium-transporting ATPase OS=Setaria | Pgl_GLEAN_10020375 | 1.95 | 2.45 | 2.30 |  |  |  |
|  | Pgl_GLEAN_10027824 | 2.36 | 2.48 | 2.62 |  |  |  |
|  | Pgl_GLEAN_10036166 | 2.19 | 3.15 | 2.45 |  |  |  |
| HATPase_c domain-containing protein | Pgl_GLEAN_10018470 | 6.08 | 6.48 | 6.24 |  |  |  |
| Ferritin OS=Setaria italica OX=4555 GN=101778084 | Pgl_GLEAN_10002079 |  | -2.53 | -2.27 | -2.18 | -1.92 |  |
| Vacuolar cation/proton exchanger | Pgl_GLEAN_10034895 |  | 1.89 | 2.16 |  |  |  |
| Solute carrier family 40 protein OS=Setaria | Pgl_GLEAN_10004868 |  | -1.71 | -2.01 |  |  |  |
| Protein ZINC INDUCED FACILITATOR-LIKE | Pgl_GLEAN_10025753 |  | 2.40 |  |  |  | -2.94 |
| Zinc transporter 5 OS=Triticum urartu | Pgl_GLEAN_10036448 |  | -2.67 | -1.61 | -2.22 |  |  |
| Potassium transporter OS=Setaria italica | Pgl_GLEAN_10018631 |  | 2.22 |  |  |  |  |
| Uncharacterized protein OS=Setaria | Pgl_GLEAN_10021386 |  | 1.79 |  |  |  |  |
| Uncharacterized protein OS=Setaria italica | Pgl_GLEAN_10005377 |  |  |  | 2.88 |  |  |
| Uncharacterized protein OS=Setaria italica | Pgl_GLEAN_10013687 |  |  | -2.66 | -2.27 | -3.70 |  |
| Uncharacterized protein OS=Setaria italica | Pgl_GLEAN_10028548 | 7.61 | 7.94 | 7.72 |  |  |  |

| **Table S3: List of DEGs related photosynthesis pathway identified in response to –Fe, –Zn, –Fe–Zn stress in leaf tissue** | | | | | | | |
| --- | --- | --- | --- | --- | --- | --- | --- |
|  |  |  |  |  |  |  |  |
| **Description** | **Gene IDs** | **Leaf+Fe+Zn Leaf+Fe–Zn** | **Leaf+Fe+Zn Leaf–Fe+Zn** | **Leaf+Fe+Zn Leaf–Fe–Zn** | **Leaf+Fe–Zn Leaf–Fe+Zn** | **Leaf+Fe–Zn Leaf–Fe–Zn** | **Leaf–Fe+Zn Leaf–Fe–Zn** |
| Sucrose synthase OS=Panicum miliaceum | Pgl_GLEAN_10032110 | 2.49 | 2.54 | 2.51 |  |  |  |
| Starch synthase, chloroplastic/amyloplastic | Pgl_GLEAN_10033473 | 5.11 | 5.26 | 5.24 |  |  |  |
| Photosystem II CP47 reaction center | Pgl_GLEAN_10001458 |  | 3.62 | 3.16 | 3.13 | 2.61 |  |
| Thioredoxin-like 1-3, chloroplastic | Pgl_GLEAN_10002004 | -1.97 | -2.53 | -3.24 |  |  |  |
| Ubiquinol oxidase OS=Setaria viridis | Pgl_GLEAN_10004208 | 2.09 | 1.84 | 2.02 |  |  |  |
| NAD(P)H dehydrogenase (quinone) | Pgl_GLEAN_10026408 | 2.32 | 2.14 | 2.01 |  |  |  |
|  | Pgl_GLEAN_10009527 | 8.62 | 9.54 | 9.49 |  |  |  |
| NADH:ubiquinone reductase (non-electrogenic) | Pgl_GLEAN_10002501 |  | 1.71 |  |  |  |  |
| Carbonic anhydrase OS=Panicum | Pgl_GLEAN_10007471 |  | -1.90 |  |  |  |  |
| ATP synthase subunit alpha OS=Aegilops | Pgl_GLEAN_10024475 |  | 2.93 | 3.38 |  |  |  |
| Photosystem I P700 chlorophyll a apoprotein | Pgl_GLEAN_10022154 |  |  | 2.21 |  | 1.93 | 1.66 |
| Thioredoxin reductase OS=Setaria italica | Pgl_GLEAN_10022875 |  |  | -1.60 |  |  |  |
| Chlorophyll a-b binding protein, chloroplastic | Pgl_GLEAN_10026426 |  |  | -2.05 |  |  |  |
| Ribulose bisphosphate carboxylase | Pgl_GLEAN_10027288 |  |  |  | 2.19 | 2.11 |  |

| **Table S4: List of DEGs related to carbohydrate metabolism identified in response to –Fe, –Zn, –Fe–Zn stress in leaf tissue** | | | | | | | | | | | | | | | | |
| --- | --- | --- | --- | --- | --- | --- | --- | --- | --- | --- | --- | --- | --- | --- | --- | --- |
|  | |  | |  | |  | |  | |  | |  | |  | | |
| **Description** | | **Gene IDs** | | **Leaf+Fe+Zn Leaf+Fe–Zn** | | **Leaf+Fe+Zn Leaf–Fe+Zn** | | **Leaf+Fe+Zn Leaf–Fe–Zn** | | **Leaf+Fe–Zn Leaf–Fe+Zn** | | **Leaf+Fe–Zn Leaf–Fe–Zn** | | **Leaf–Fe+Zn Leaf–Fe–Zn** | | |
| Glyceraldehyde-3-phosphate dehydrogenase | | Pgl_GLEAN_10000341 | | 2.46 | | 3.22 | | 2.77 | |  | |  | |  | | |
| Phosphoglycerate mutase (2,3-diphosphoglycerate-independent) | | Pgl_GLEAN_10009672 | | 4.44 | | 4.50 | | 4.72 | |  | |  | |  | | |
| Pyrophosphate--fructose 6-phosphate | | Pgl_GLEAN_10011778 | | 2.36 | | 3.15 | | 2.91 | |  | |  | |  | | |
| ATP-dependent 6-phosphofructokinase | | Pgl_GLEAN_10012726 | | 3.37 | | 3.54 | | 3.72 | |  | |  | |  | | |
|  | | Pgl_GLEAN_10034681 | | 5.44 | | 5.72 | | 5.73 | |  | |  | |  | | |
| Pyruvate decarboxylase 1 OS=Dichanthelium | | Pgl_GLEAN_10026657 | | 8.21 | | 8.42 | | 8.43 | |  | |  | |  | | |
| Pyruvate dehydrogenase E1 component | | Pgl_GLEAN_10013093 | | 2.74 | | 4.13 | | 2.91 | |  | |  | |  | | |
| Pyruvate kinase OS=Panicum hallii | | Pgl_GLEAN_10032591 | | 2.19 | | 2.36 | | 2.36 | |  | |  | |  | | |
| Thiamine pyrophosphokinase OS=Setaria | | Pgl_GLEAN_10022726 | | 1.90 | | 2.26 | | 2.00 | |  | |  | |  | | |
|  | |  | |  | |  | |  | |  | |  | |  | | |
| Glycerol-3-phosphate dehydrogenase | | Pgl_GLEAN_10037186 | | 2.56 | | 2.83 | | 2.99 | |  | |  | |  | | |
| APO protein 4, mitochondrial OS=Panicum | | Pgl_GLEAN_10004075 | | 2.15 | | 2.19 | | 2.39 | |  | |  | |  | | |
| Aldose 1-epimerase OS=Setaria | | Pgl_GLEAN_10021973 | |  | | 7.23 | |  | | 2.75 | |  | |  | | |
| Succinate dehydrogenase [ubiquinone] flavoprotein | | Pgl_GLEAN_10022824 | |  | | 2.83 | |  | | 1.98 | |  | |  | | |
| Phosphoenolpyruvate carboxylase OS=Setaria | | Pgl_GLEAN_10026714 | |  | | 1.99 | |  | |  | |  | |  | | |
|  | | Pgl_GLEAN_10027484 | |  | | 2.07 | |  | |  | |  | |  | | |
| Mitochondrial phosphate carrier | | Pgl_GLEAN_10035565 | |  | | 1.57 | |  | |  | |  | |  | | |
|  | | Pgl_GLEAN_10003107 | |  | | 2.74 | |  | |  | |  | |  | | |
| Aconitate hydratase OS=Panicum | | Pgl_GLEAN_10037028 | |  | | 2.66 | |  | |  | |  | |  | | |
| Malic enzyme OS=Setaria viridis | | Pgl_GLEAN_10016602 | | -3.10 | |  | |  | |  | |  | |  | | |
| Isocitrate dehydrogenase [NADP] OS=Setaria | | Pgl_GLEAN_10018367 | |  | |  | | -1.64 | |  | |  | |  | | |
| Alcohol dehydrogenase 1 OS=Cenchrus americanus | | Pgl_GLEAN_10015813 | | 3.61 | | 3.61 | | 3.61 | |  | |  | |  | | |
| Nitrate reductase OS=Panicum miliaceum | | Pgl_GLEAN_10033153 | |  | | -1.80 | |  | | -1.80 | |  | | 1.59 | | |
| Ribulose-phosphate 3-epimerase OS=Setaria | | Pgl_GLEAN_10033298 | | -2.35 | |  | |  | | 2.78 | |  | |  | | |
|  | | | | | | | | | | | | | | | | |
| **Table S5: List of DEGs related to plant hormones biosynthesis identified in response to –Fe, –Zn, –Fe–Zn stress in leaf tissue** | | | | | | | | | | | | | | | |  |
|  | |  | |  | |  | |  | |  | |  | |  | |  |
| **Description** | | **Gene IDs** | | **Leaf+Fe+Zn Leaf+Fe–Zn** | | **Leaf+Fe+Zn Leaf–Fe+Zn** | | **Leaf+Fe+Zn Leaf–Fe–Zn** | | **Leaf+Fe–Zn Leaf–Fe+Zn** | | **Leaf+Fe–Zn Leaf–Fe–Zn** | | **Leaf–Fe+Zn Leaf–Fe–Zn** | |  |
| Indole-3-glycerol-phosphate synthase OS=Setaria | | Pgl_GLEAN_10007029 | | 1.66 | | 2.33 | | 2.29 | |  | |  | |  | |  |
| Cytokinin dehydrogenase OS=Setaria italica | | Pgl_GLEAN_10020916 | | 3.75 | | 4.08 | | 3.96 | |  | |  | |  | |  |
| Cytokinin riboside 5'-monophosphate phosphoribohydrolase | | Pgl_GLEAN_10009839 | | 2.11 | | 2.16 | | 2.41 | |  | |  | |  | |  |
| 1-aminocyclopropane-1-carboxylate | | Pgl_GLEAN_10012661 | | 2.82 | | 2.89 | | 3.03 | |  | |  | |  | |  |
| AP2-like ethylene-responsive transcription | | Pgl_GLEAN_10018408 | | 4.14 | | 5.43 | | 4.33 | |  | |  | |  | |  |
| Terpene synthase TPS25 OS=Setaria | | Pgl_GLEAN_10021117 | | 5.93 | | 6.41 | | 6.27 | |  | |  | |  | |  |
|  | | Pgl_GLEAN_10022268 | | 3.29 | | 3.51 | | 3.51 | |  | |  | |  | |  |
| Auxin response factor OS=Setaria | | Pgl_GLEAN_10031878 | | 2.15 | | 2.26 | | 2.44 | |  | |  | |  | |  |
|  | | Pgl_GLEAN_10036550 | | 2.53 | | 2.74 | | 2.83 | |  | |  | |  | |  |
|  | | Pgl_GLEAN_10002173 | | 1.81 | | 2.32 | | 2.02 | |  | |  | |  | |  |
| Auxin-responsive protein OS=Setaria | | Pgl_GLEAN_10013170 | | 7.45 | | 7.77 | |  | |  | |  | |  | |  |
| Cytochrome P450 87A3 OS=Dichanthelium | | Pgl_GLEAN_10002931 | | 3.40 | | 3.36 | | 3.48 | |  | |  | |  | |  |
| Auxin response factor 23 OS=Dichanthelium | | Pgl_GLEAN_10001638 | | 2.59 | |  | |  | |  | |  | |  | |  |
| ABA receptor SnRK2.9 OS=Setaria | | Pgl_GLEAN_10021784 | |  | |  | | 1.84 | |  | |  | |  | |  |
| Auxin efflux carrier component | | Pgl_GLEAN_10015150 | |  | |  | | 2.50 | |  | |  | |  | |  |

| **Table S6: List of DEGs related to other cellular pathways identified in response to –Fe, –Zn, –Fe–Zn stress in leaf tissue** | | | | | | | | |
| --- | --- | --- | --- | --- | --- | --- | --- | --- |
|  |  |  |  |  |  |  |  |  |
| **Description** | **Gene IDs** | **Leaf+Fe+Zn Leaf+Fe–Zn** | **Leaf+Fe+Zn Leaf–Fe+Zn** | **Leaf+Fe+Zn Leaf–Fe–Zn** | **Leaf+Fe–Zn Leaf–Fe+Zn** | **Leaf+Fe–Zn Leaf–Fe–Zn** | **Leaf–Fe+Zn Leaf–Fe–Zn** |  |
| Xyloglucan endotransglucosylase/hydrolase | Pgl_GLEAN_10016859 | 2.18 | 2.54 | 2.37 |  |  |  |  |
|  | Pgl_GLEAN_10032011 | 4.74 | 5.60 | 5.29 |  |  |  |  |
|  | Pgl_GLEAN_10033971 | 2.52 | 2.33 | 2.67 |  |  |  |  |
|  | Pgl_GLEAN_10037906 | 6.84 | 7.07 | 7.15 |  |  |  |  |
| Trehalose 6-phosphate phosphatase | Pgl_GLEAN_10035143 | 1.92 | 3.05 | 2.20 |  |  |  |  |
| Cellulose synthase-like protein | Pgl_GLEAN_10028813 | 7.53 | 7.70 | 7.76 |  |  |  |  |
| Expansin OS=Setaria viridis OX=4556 | Pgl_GLEAN_10018528 | 2.78 | 3.12 | 3.25 |  |  |  |  |
|  | Pgl_GLEAN_10014257 | 4.37 | 4.64 | 4.45 |  |  |  |  |
| Cinnamoyl-CoA reductase 1-like | Pgl_GLEAN_10008961 | 2.37 | 2.44 | 2.47 |  |  |  |  |
| O-fucosyltransferase family protein | Pgl_GLEAN_10030768 | 3.04 | 3.49 | 3.41 |  |  |  |  |
|  | Pgl_GLEAN_10032062 | 4.37 | 4.42 | 4.64 |  |  |  |  |
|  | Pgl_GLEAN_10015641 | 1.78 | 2.00 | 1.90 |  |  |  |  |
|  | Pgl_GLEAN_10033586 | 2.49 | 2.45 | 2.63 |  |  |  |  |
| S-acyltransferase OS=Panicum hallii | Pgl_GLEAN_10006928 | 4.28 | 4.89 | 4.78 |  |  |  |  |
|  | Pgl_GLEAN_10022503 | 4.73 | 6.69 | 5.26 |  |  |  |  |
|  | Pgl_GLEAN_10025986 | 4.03 | 4.09 | 4.30 |  |  |  |  |
| 3-ketoacyl-CoA synthase OS=Setaria | Pgl_GLEAN_10012220 | 3.77 | 2.71 | 3.24 |  |  |  |  |
|  | Pgl_GLEAN_10029153 | 5.59 | 6.76 | 6.32 |  |  |  |  |
|  | Pgl_GLEAN_10020274 | 1.96 | 2.14 | 2.43 |  |  |  |  |
|  | Pgl_GLEAN_10028162 | 6.82 | 7.53 | 7.00 |  |  |  |  |
|  | Pgl_GLEAN_10031126 | 3.83 | 4.04 | 4.08 |  |  |  |  |
|  | Pgl_GLEAN_10036173 | 6.30 | 6.73 | 6.45 |  |  |  |  |
| Diacylglycerol kinase OS=Panicum | Pgl_GLEAN_10024874 | 2.96 | 3.64 | 3.26 |  |  |  |  |
|  | Pgl_GLEAN_10032961 | 2.41 | 3.50 | 2.74 |  |  |  |  |
| Phospholipase D OS=Panicum miliaceum | Pgl_GLEAN_10007182 | 2.02 | 2.08 | 2.18 |  |  |  |  |
|  | Pgl_GLEAN_10028562 | 4.78 | 5.10 | 4.95 |  |  |  |  |
|  | Pgl_GLEAN_10020424 | 3.89 | 5.14 | 4.04 |  |  |  |  |
| Diacylglycerol O-acyltransferase | Pgl_GLEAN_10008628 | 4.80 | 4.99 | 4.88 |  |  |  |  |
| Peroxidase OS=Setaria italica | Pgl_GLEAN_10002230 | 7.38 | 8.77 | 8.06 |  |  |  |  |
|  | Pgl_GLEAN_10015996 | 3.11 | 3.66 | 3.49 |  |  |  |  |
|  | Pgl_GLEAN_10024220 | 3.36 | 3.39 | 3.66 |  |  |  |  |
| Putative thaumatin domain family | Pgl_GLEAN_10006585 | 4.56 | 5.55 | 5.14 |  |  |  |  |
| Dirigent protein OS=Panicum hallii | Pgl_GLEAN_10007613 | 2.75 | 2.59 | 3.08 |  |  |  |  |
|  | Pgl_GLEAN_10004363 | 2.51 | 3.03 | 2.40 |  |  |  |  |
| Mitogen-activated protein kinase | Pgl_GLEAN_10018819 | 1.66 | 1.79 | 1.78 |  |  |  |  |
| NBS-LRR disease resistance protein homologue | Pgl_GLEAN_10033214 | 1.94 | 1.88 | 2.07 |  |  |  |  |
| Serine/threonine-protein kinase | Pgl_GLEAN_10020329 | 2.25 | 2.53 | 2.78 |  |  |  |  |
|  | Pgl_GLEAN_10022079 | 2.83 | 2.93 | 2.94 |  |  |  |  |
| Serine/threonine protein phosphatase | Pgl_GLEAN_10015900 | 3.05 | 3.43 | 3.36 |  |  |  |  |
|  | Pgl_GLEAN_10033509 | 2.19 | 1.92 | 2.30 |  |  |  |  |
| MLO-like protein OS=Setaria viridis | Pgl_GLEAN_10001581 | 2.50 | 2.86 | 2.84 |  |  |  |  |
| Superoxide dismutase OS=Zea mays | Pgl_GLEAN_10001023 | -3.17 |  |  |  |  |  |  |

| **Table S7: List of DEGs related to Fe, Zn uptake and transport identified in response to –Fe, –Zn, –Fe–Zn stresses in root tissue** | | | | | | | |
| --- | --- | --- | --- | --- | --- | --- | --- |
|  |  |  |  |  |  |  |  |
| **Description** | **Gene IDs** | **Root+Fe+Zn Root+Fe–Zn** | **Root+Fe+Zn Root–Fe+Zn** | **Root+Fe+Zn Root–Fe–Zn** | **Root+Fe–Zn Root–Fe+Zn** | **Root+Fe–Zn Root–Fe–Zn** | **Root–Fe+Zn Root–Fe–Zn** |
| Uncharacterized protein OS=Setaria | Pgl_GLEAN_10001420 | -2.10 | -3.04 | -2.17 |  |  |  |
| Copper transporter OS=Setaria | Pgl_GLEAN_10021313 | 1.90 |  | -2.67 | -1.51 | -4.53 | -3.11 |
| MYB transcription factor OS=Saccharum | Pgl_GLEAN_10009281 | 2.46 |  | -2.87 | -2.77 | -5.16 | -2.56 |
|  | Pgl_GLEAN_10019982 | 3.20 |  |  |  | -6.51 | -5.51 |
| HMA domain-containing protein OS=Setaria | Pgl_GLEAN_10030699 | 1.69 |  | -2.25 | -2.19 | -3.83 |  |
| 2-oxoglutarate (2OG) and Fe(II)-dependent | Pgl_GLEAN_10029465 |  | -1.64 |  |  |  |  |
| ABC transporter G family member | Pgl_GLEAN_10032979 |  |  | 1.92 |  | 2.14 | 1.63 |
| Vacuolar cation/proton exchanger OS=Setaria | Pgl_GLEAN_10036960 |  |  | -2.11 |  | -3.44 | -2.51 |
| Solute carrier family 40 protein OS=Setaria | Pgl_GLEAN_10004868 |  |  | -2.63 | -2.65 | -4.24 |  |
| 1,2-dihydroxy-3-keto-5-methylthiopentene | Pgl_GLEAN_10038575 |  |  | 1.82 |  | 3.52 |  |
| Oligopeptide transporter 3 OS=Zea | Pgl_GLEAN_10016104 |  |  | 1.65 |  |  |  |
| Adenine phosphoribosyltransferase | Pgl_GLEAN_10012911 |  |  |  | 2.24 | 2.04 |  |
|  | Pgl_GLEAN_10029973 | 1.82 |  |  | -1.73 | -2.31 |  |
|  | Pgl_GLEAN_10008124 | 1.58 |  |  |  | -2.39 |  |
| Potassium transporter OS=Setaria italica | Pgl_GLEAN_10005900 |  |  | -2.23 |  | -2.63 | -2.73 |
|  | Pgl_GLEAN_10007434 |  |  | -1.88 |  | -2.24 | -2.29 |
|  | Pgl_GLEAN_10031148 |  |  | -1.63 |  | -2.62 | -2.13 |
|  | Pgl_GLEAN_10002079 |  |  |  | -2.06 | -2.48 |  |
| Ferritin OS=Setaria italica OX=4555 GN=101778084 | Pgl_GLEAN_10036448 |  |  |  |  | -1.73 |  |
| Zinc transporter 5 OS=Triticum urartu | Pgl_GLEAN_10010955 |  |  | -1.82 |  | -2.98 | -2.22 |
| Magnesium transporter OS=Panicum | Pgl_GLEAN_10024237 |  | 2.28 | 1.96 | 2.98 | 2.58 |  |
| Aldo_ket_red domain-containing | Pgl_GLEAN_10015686 |  | 3.62 | 2.13 | 4.20 | 2.60 |  |
| Uncharacterized protein OS=Setaria | Pgl_GLEAN_10002050 |  |  |  |  | -2.39 |  |
| Aspartate aminotransferase OS=Panicum | Pgl_GLEAN_10006455 |  |  |  | 1.93 |  |  |
| Ribose-5-phosphate isomerase OS=Setaria | Pgl_GLEAN_10033869 | 1.74 |  |  |  | -3.00 | -1.76 |

| **Table S8: List of DEGs related to photosynthesis identified in response to –Fe, –Zn, –Fe–Zn stresses in root tissue** | | | | | | | |
| --- | --- | --- | --- | --- | --- | --- | --- |
|  |  |  |  |  |  |  |  |
| **Description** | **Gene IDs** | **Root+Fe+Zn Root+Fe–Zn** | **Root+Fe+Zn Root–Fe+Zn** | **Root+Fe+Zn Root–Fe–Zn** | **Root+Fe–Zn Root–Fe+Zn** | **Root+Fe–Zn Root–Fe–Zn** | **Root–Fe+Zn Root–Fe–Zn** |
| Magnesium chelatase OS=Setaria | Pgl_GLEAN_10012641 | 1.98 |  | -3.07 |  | -4.93 | -3.87 |
| Mg-protoporphyrin IX chelatase OS=Setaria | Pgl_GLEAN_10038264 | 1.82 |  | -2.14 |  | -3.86 | -2.64 |
| Chlorophyll a-b binding protein, chloroplastic | Pgl_GLEAN_10013085 | 2.68 |  | -4.06 |  | -6.59 | -5.34 |
| NADPH-protochlorophyllide oxidoreductase | Pgl_GLEAN_10028726 | 2.72 |  | -4.46 | -1.90 | -7.03 | -5.26 |
| Plastocyanin OS=Panicum hallii | Pgl_GLEAN_10001189 | 2.69 |  | -3.95 |  | -6.49 | -5.60 |
| Thioredoxin H-type 2-like OS=Panicum | Pgl_GLEAN_10005672 | 2.89 |  | -5.29 |  | -7.82 | -6.40 |
| Ferredoxin OS=Sorghum bicolor OX=4558 | Pgl_GLEAN_10003612 | 2.36 |  | -3.86 |  | -6.14 | -5.12 |
| Ferredoxin--NADP reductase, chloroplastic | Pgl_GLEAN_10003660 | 1.69 |  | -3.91 |  | -4.57 | -3.56 |
| Plastoquinol--plastocyanin reductase OS=Setaria | Pgl_GLEAN_10014480 | 2.26 |  | -3.25 |  | -5.37 | -4.15 |
| Photosystem I P700 chlorophyll a apoprotein | Pgl_GLEAN_10022154 | 2.56 | 2.37 | -2.07 |  | -4.53 | -4.41 |
| Uncharacterized protein OS=Setaria italica | Pgl_GLEAN_10020492 | 2.42 |  |  |  |  |  |
| Photosystem II 10 kDa polypeptide, chloroplastic | Pgl_GLEAN_10023088 | 2.46 |  | -4.62 |  | -6.94 | -5.73 |
| PSII 6.1 kDa protein OS=Setaria italica | Pgl_GLEAN_10019587 | 2.78 |  | -2.86 |  |  |  |
| PSI subunit V OS=Panicum hallii | Pgl_GLEAN_10020246 | 2.78 |  | -4.28 |  |  |  |
| Cytochrome c oxidase subunit OS=Setaria | Pgl_GLEAN_10025838 | -1.53 |  |  |  | 1.56 |  |
| Cytochrome c OS=Zea mays OX=4577 PE=2 | Pgl_GLEAN_10022444 | -2.03 |  |  |  | 1.72 |  |
| Ribulose bisphosphate carboxylase | Pgl_GLEAN_10027288 | 1.83 | 1.67 | -2.62 |  | -4.42 | -4.30 |
| Ribose-5-phosphate isomerase OS=Setaria | Pgl_GLEAN_10033869 | 1.74 |  |  |  | -3.00 |  |
| NAD(P)H dehydrogenase (quinone) | Pgl_GLEAN_10003907 | -2.81 | -2.42 |  |  |  |  |
| Ubiquinol oxidase OS=Setaria viridis | Pgl_GLEAN_10004207 | -2.50 | -1.67 |  |  |  |  |
| Photosystem II CP47 reaction center | Pgl_GLEAN_10001458 |  | 1.61 | -1.64 |  | -2.75 | -3.22 |
| Carbonic anhydrase OS=Setaria | Pgl_GLEAN_10007313 | 2.03 |  | -2.31 |  | -4.20 | -3.16 |
| Starch synthase, chloroplastic/amyloplastic | Pgl_GLEAN_10033473 |  |  | -2.01 |  | -2.42 | -2.61 |
| Sucrose synthase OS=Setaria viridis | Pgl_GLEAN_10008136 |  |  | -1.64 |  | -2.13 | -2.04 |
| Cytochrome b-c1 complex subunit 6 OS=Oryza | Pgl_GLEAN_10002443 | -1.65 |  |  | 1.76 | 1.86 |  |
| Thioredoxin reductase OS=Setaria italica | Pgl_GLEAN_10022875 |  |  |  | -1.80 |  |  |
| ATP synthase subunit d, mitochondrial | Pgl_GLEAN_10017270 |  |  |  | 1.54 |  |  |
| Ferrochelatase OS=Setaria viridis | Pgl_GLEAN_10011603 |  |  |  |  | -2.53 | -1.54 |
| NAD(P)H dehydrogenase subunit | Pgl_GLEAN_10013683 | 1.79 |  |  |  | -2.56 | -2.00 |
| Iron-sulfur assembly protein IscA | Pgl_GLEAN_10034808 |  |  |  |  | -1.92 |  |
| Photolyase/cryptochrome alpha/beta | Pgl_GLEAN_10005600 |  |  |  |  | -1.59 | -1.64 |
|  | Pgl_GLEAN_10008988 |  |  |  |  | -1.88 |  |

| **Table S9: List of DEGs related to carbohydrate metabolism identified in response to –Fe, –Zn, –Fe–Zn stresses in root tissue** | | | | | | | |
| --- | --- | --- | --- | --- | --- | --- | --- |
|  |  |  |  |  |  |  |  |
| **Description** | **Gene IDs** | **Root+Fe+Zn Root+Fe–Zn** | **Root+Fe+Zn Root–Fe+Zn** | **Root+Fe+Zn Root–Fe–Zn** | **Root+Fe–Zn Root–Fe+Zn** | **Root+Fe–Zn Root–Fe–Zn** | **Root–Fe+Zn Root–Fe–Zn** |
| D-3-phosphoglycerate dehydrogenase OS=Sorghum | Pgl_GLEAN_10010057 | 2.46 |  | -2.84 | -2.04 | -5.18 | -3.27 |
| Phosphoglycerate kinase OS=Setaria italica | Pgl_GLEAN_10035773 | 2.08 |  | -1.74 |  | -3.72 | -2.64 |
|  | Pgl_GLEAN_10022839 | 1.55 |  | -1.51 |  | -2.97 | -1.81 |
| Fructose-bisphosphate aldolase OS=Setaria | Pgl_GLEAN_10024933 | 2.10 |  | -3.75 |  | -5.72 | -3.87 |
| Glyceraldehyde-3-phosphate dehydrogenase | Pgl_GLEAN_10037675 | 2.42 |  |  |  | -6.85 | -5.35 |
| Phosphoenolpyruvate carboxylase OS=Cenchrus | Pgl_GLEAN_10033512 | 2.22 |  |  |  | -7.06 | -5.83 |
| Phosphoenolpyruvate carboxykinase | Pgl_GLEAN_10029931 | 2.34 |  | -3.69 |  | -5.86 | -4.53 |
| Ribose-5-phosphate isomerase OS=Setaria | Pgl_GLEAN_10033869 | 1.74 |  |  |  | -3.00 | -1.76 |
| Phosphoglucomutase | Pgl_GLEAN_10036106 | 2.44 |  |  | -1.97 | -3.70 | -1.85 |
| Pyruvate | Pgl_GLEAN_10001175 |  |  | -4.53 | -2.10 | -6.72 | -4.74 |
| Pyruvate dehydrogenase E1 component | Pgl_GLEAN_10030611 |  |  | -1.99 |  | -2.36 | -2.55 |
| Phosphoglycerate mutase | Pgl_GLEAN_10009672 |  |  | -1.72 |  |  | -2.30 |
| Isocitrate lyase OS=Setaria italica OX=4555 | Pgl_GLEAN_10010163 |  |  | -1.82 |  | -2.20 | -1.74 |
| Aconitate hydratase OS=Panicum | Pgl_GLEAN_10037028 |  |  | -1.65 |  | -2.09 | -2.30 |
| D-fructose-1,6-bisphosphate 1-phosphohydrolase | Pgl_GLEAN_10017683 |  |  | -2.46 |  | -3.82 | -2.95 |
| Glucose-6-phosphate 1-dehydrogenase | Pgl_GLEAN_10034350 | 2.12 |  |  | -1.91 | -3.23 |  |
| L-lactate dehydrogenase OS=Setaria italica | Pgl_GLEAN_10018121 | -1.69 |  |  |  | 1.75 |  |

| **Table S10: List of DEGs related to plant hormones biosynthesis identified in response to –Fe, –Zn, –Fe–Zn stresses in root tissue** | | | | | | | |
| --- | --- | --- | --- | --- | --- | --- | --- |
|  |  |  |  |  |  |  |  |
| **Description** | **Gene IDs** | **Root+Fe+Zn Root+Fe–Zn** | **Root+Fe+Zn Root–Fe+Zn** | **Root+Fe+Zn Root–Fe–Zn** | **Root+Fe–Zn Root–Fe+Zn** | **Root+Fe–Zn Root–Fe–Zn** | **Root–Fe+Zn Root–Fe–Zn** |
| Auxin-responsive protein SAUR36 | Pgl_GLEAN_10002746 | 2.95 | 1.95 |  |  | -4.54 | -3.58 |
| 15-cis-phytoene synthase OS=Sorghum bicolor | Pgl_GLEAN_10005849 | -2.06 |  | -1.86 |  | 2.55 |  |
| Auxin-responsive protein OS=Setaria italica | Pgl_GLEAN_10017400 | -2.23 |  | -1.87 | 2.31 | 2.61 |  |
| Cytokinin riboside 5'-monophosphate | Pgl_GLEAN_10016650 | 1.93 |  | -2.18 |  |  | -2.87 |
| Cytokinin dehydrogenase OS=Setaria italica | Pgl_GLEAN_10024669 | -2.13 |  |  | 2.31 |  | -2.45 |
| Cytokinin-N-glucosyltransferase | Pgl_GLEAN_10036480 | -2.34 |  |  |  |  |  |
| Cytochrome P450 94A1 OS=Dichanthelium | Pgl_GLEAN_10014404 | 1.67 |  |  |  |  |  |
| Cytochrome P450 76C4 OS=Panicum | Pgl_GLEAN_10038156 | 1.61 |  |  |  |  |  |
| Cytochrome P450 72A14 OS=Dichanthelium | Pgl_GLEAN_10032881 | 2.36 |  |  |  |  |  |
| Auxin efflux carrier component OS=Setaria | Pgl_GLEAN_10018536 |  | -1.59 | -1.59 |  |  | 1.50 |
| Auxin response factor OS=Setaria italica | Pgl_GLEAN_10035663 |  | -2.16 |  |  |  | 2.01 |
| Terpene cyclase/mutase family | Pgl_GLEAN_10038495 |  |  | -1.72 |  | -2.68 | -1.75 |
|  | Pgl_GLEAN_10012661 |  |  | -1.88 | 2.90 | 2.25 | -2.51 |
| 1-aminocyclopropane-1-carboxylate | Pgl_GLEAN_10021117 |  |  | -2.05 |  |  | -2.72 |
| Terpene synthase TPS25 OS=Setaria | Pgl_GLEAN_10018525 | 2.24 |  | -2.92 |  | -5.03 | -3.68 |

| **Table S11: List of DEGs related to other cellular pathways identified in response to –Fe, –Zn, –Fe–Zn stresses in root tissue** | | | | | | | |
| --- | --- | --- | --- | --- | --- | --- | --- |
|  |  |  |  |  |  |  |  |
| **Description** | **Gene IDs** | **Root+Fe+Zn Root+Fe–Zn** | **Root+Fe+Zn Root–Fe+Zn** | **Root+Fe+Zn Root–Fe–Zn** | **Root+Fe–Zn Root–Fe+Zn** | **Root+Fe–Zn Root–Fe–Zn** | **Root–Fe+Zn Root–Fe–Zn** |
| Xyloglucan endotransglucosylase/hydrolase | Pgl_GLEAN_10017938 | 1.69 |  | -1.71 |  | -3.29 | -1.94 |
|  | Pgl_GLEAN_10020386 | 2.06 |  | -3.42 | -2.00 | -5.31 | -3.46 |
| Trehalose 6-phosphate phosphatase | Pgl_GLEAN_10012421 | -1.94 | -2.12 |  |  |  | 1.67 |
| Expansin OS=Setaria viridis OX=4556 | Pgl_GLEAN_10013160 | 2.07 | 2.45 | 1.90 | 5.00 | 4.39 |  |
| 3-ketoacyl-CoA synthase OS=Setaria italica | Pgl_GLEAN_10038530 | 1.63 |  | -2.85 |  | -4.34 | -3.77 |
| Phospholipase A1 OS=Setaria viridis | Pgl_GLEAN_10024567 | 2.28 |  | -2.02 | -1.72 | -4.20 | -2.61 |
| Diacylglycerol O-acyltransferase OS=Setaria | Pgl_GLEAN_10021320 | 1.63 |  | -2.21 |  | -3.72 | -2.79 |
|  | Pgl_GLEAN_10004086 | 1.99 |  |  | -2.65 | -3.80 |  |
| Diacylglycerol kinase OS=Panicum | Pgl_GLEAN_10029548 | -1.90 |  |  |  | 1.89 |  |
| Peroxidase OS=Setaria viridis | Pgl_GLEAN_10035363 | 1.76 |  |  |  | -3.14 | -1.95 |
|  | Pgl_GLEAN_10004076 | -3.19 |  |  |  | 3.77 |  |
|  | Pgl_GLEAN_10011921 | -2.70 | -2.80 |  |  | 2.74 | 2.84 |
|  | Pgl_GLEAN_10015223 | -2.79 | -2.09 |  |  | 2.90 | 2.24 |
|  | Pgl_GLEAN_10024308 | -2.05 |  |  |  | 2.07 |  |
|  | Pgl_GLEAN_10024312 | -1.83 |  |  |  | 1.77 |  |
|  | Pgl_GLEAN_10022720 | -1.72 |  |  |  | 1.71 |  |
|  | Pgl_GLEAN_10017247 | -2.18 |  |  |  | 2.34 |  |
|  | Pgl_GLEAN_10038421 | -2.64 |  |  |  |  |  |
|  | Pgl_GLEAN_10011922 | -2.36 |  |  |  | 2.57 |  |
|  | Pgl_GLEAN_10015993 | -2.16 |  |  | 2.60 | 2.82 |  |
|  | Pgl_GLEAN_10006484 | -3.49 | -2.56 |  |  | 4.11 | 3.21 |
|  | Pgl_GLEAN_10009714 | -1.73 |  |  |  | 2.41 |  |
|  | Pgl_GLEAN_10014871 | 1.65 |  |  |  | -3.82 | -3.14 |
| Dirigent protein OS=Triticum aestivum | Pgl_GLEAN_10000800 | 2.66 |  |  |  | -7.09 | -5.88 |
|  | Pgl_GLEAN_10002657 | 2.37 |  |  |  | -4.79 | -4.25 |
|  | Pgl_GLEAN_10038229 | -3.87 |  |  |  | 3.52 |  |
|  | Pgl_GLEAN_10033001 | -2.55 |  |  | 3.02 | 2.05 |  |
|  | Pgl_GLEAN_10007613 | -1.58 |  |  |  |  |  |
|  | Pgl_GLEAN_10022602 | -4.28 |  | -1.76 |  |  |  |
|  | Pgl_GLEAN_10000798 | 3.08 | 3.04 |  |  | -7.81 | -7.85 |
|  | Pgl_GLEAN_10024110 | 2.53 | 2.64 |  |  | -2.45 | -2.61 |
| Superoxide dismutase OS=Setaria italica | Pgl_GLEAN_10037098 | 1.68 |  |  | -1.86 | -2.99 |  |
| Protein-serine/threonine phosphatase | Pgl_GLEAN_10036943 | 2.29 | -1.76 | -1.65 | -2.00 | -3.83 | -1.94 |
| Protein-serine/threonine kinase OS=Setaria | Pgl_GLEAN_10004592 | 1.90 | -2.66 | -2.21 |  | -3.97 | -2.50 |
| L-ascorbate peroxidase OS=Setaria italica | Pgl_GLEAN_10006755 | 1.86 |  | -2.10 |  | -3.85 | -2.52 |
| Catalase OS=Sorghum bicolor OX=4558 | Pgl_GLEAN_10018073 |  |  |  |  | -2.19 |  |
| Cellulose synthase-like protein | Pgl_GLEAN_10028813 |  |  | -1.52 |  |  | -1.92 |

| **Table S12: List of DEGs related to Fe, Zn uptake and transport activity identified in response to –Fe, –Zn, –Fe–Zn stress in leaf and root comparison** | | | | | | | |
| --- | --- | --- | --- | --- | --- | --- | --- |
|  |  |  |  |  |  |  |  |
| **Description** | **Gene IDs** | **Leaf+Fe–Zn Root–Fe+Zn** | **Leaf+Fe–Zn Root–Fe–Zn** | **Leaf–Fe+Zn Root–Fe–Zn** | **Root+Fe–Zn Leaf–Fe+Zn** | **Root+Fe–Zn Leaf–Fe–Zn** | **Root–Fe+Zn Leaf–Fe–Zn** |
| Formate dehydrogenase, mitochondrial | Pgl_GLEAN_10019693 | 7.69 | 6.61 | 6.74 | 5.92 |  | -6.88 |
| Aldo_ket_red domain-containing | Pgl_GLEAN_10024237 | 4.88 | 4.50 | 4.47 | 2.98 |  | -4.33 |
| Uncharacterized protein OS=Setaria | Pgl_GLEAN_10015686 | 9.06 | 7.48 | 5.68 | 4.20 | -2.92 | -7.09 |
| ABC transporter G family member | Pgl_GLEAN_10032979 | 2.15 | 3.75 | 1.77 |  |  |  |
| Transcription factor FER-LIKE | Pgl_GLEAN_10033886 | 8.74 | 9.02 | 4.35 |  | -5.65 | -6.81 |
| 2'-deoxymugineic-acid 2'-dioxygenase | Pgl_GLEAN_10012139 | 7.24 | 8.25 | 4.52 |  |  | -4.53 |
| Heavy metal transport/detoxification | Pgl_GLEAN_10008667 | 1.99 | 1.76 | 1.83 |  |  |  |
| Calcium-transporting ATPase OS=Setaria | Pgl_GLEAN_10035767 | 2.05 | 2.39 | 2.68 |  |  | -1.67 |
| BZIP domain-containing protein OS=Setaria | Pgl_GLEAN_10038005 | 4.46 | 5.51 | 1.96 |  |  | -2.47 |
|  | Pgl_GLEAN_10019857 |  |  | 1.73 |  |  |  |
|  | Pgl_GLEAN_10036803 |  | 1.65 | 1.79 |  |  |  |
|  | Pgl_GLEAN_10021506 | 7.51 | 7.60 | 5.02 |  |  | -4.70 |
|  | Pgl_GLEAN_10002390 |  | 4.96 | 3.56 |  |  |  |
|  | Pgl_GLEAN_10007430 |  | 2.44 | 1.60 |  |  |  |
|  | Pgl_GLEAN_10015462 |  | 1.59 | 2.84 |  |  |  |
| MYB transcription factor OS=Saccharum | Pgl_GLEAN_10009281 | -3.01 | -5.40 | -5.60 | -2.77 |  | 2.02 |
|  | Pgl_GLEAN_10019982 | -2.60 | -8.22 | -8.81 |  | 1.52 | 2.63 |
| Mugineic-acid 3-dioxygenase OS=Hordeum vulgare | Pgl_GLEAN_10020996 | -2.20 | -5.48 | -3.73 |  |  |  |
| Zinc transporter 5 OS=Triticum urartu | Pgl_GLEAN_10036448 | -2.59 | -2.92 |  |  |  |  |
| Zinc transporter 3 OS=Dichanthelium | Pgl_GLEAN_10032199 | -1.58 |  |  |  |  |  |
| Protein ZINC INDUCED FACILITATOR-LIKE | Pgl_GLEAN_10025753 |  |  | -2.85 |  |  |  |
| S-adenosylmethionine synthase OS=Setaria | Pgl_GLEAN_10006489 |  |  | 1.95 |  |  |  |
| 1,2-dihydroxy-3-keto-5-methylthiopentene | Pgl_GLEAN_10038575 |  | 6.62 | 3.33 |  |  |  |
| 5-methyltetrahydropteroyltriglutamate--homocysteine | Pgl_GLEAN_10016462 |  |  | 1.90 |  |  |  |
|  | Pgl_GLEAN_10004517 |  |  | 1.59 |  |  |  |
| Uncharacterized protein OS=Setaria | Pgl_GLEAN_10003646 | -1.79 |  |  |  |  | 1.66 |
| Adenine phosphoribosyltransferase | Pgl_GLEAN_10012911 | 2.14 | 1.97 | 2.40 | 2.24 |  | -1.64 |
| Aspartate aminotransferase OS=Panicum | Pgl_GLEAN_10002050 | -2.39 | -3.43 | -2.67 |  |  | 1.58 |
| Potassium transporter OS=Setaria italica | Pgl_GLEAN_10015230 |  | 6.92 | 4.03 |  |  |  |
|  | Pgl_GLEAN_10008124 | -2.20 | -3.51 | -3.34 |  |  | 2.15 |
|  | Pgl_GLEAN_10005900 |  | -2.46 | -2.71 |  |  |  |
|  | Pgl_GLEAN_10007434 |  |  | -1.85 |  |  |  |
|  | Pgl_GLEAN_10018631 |  |  | -2.27 |  |  |  |
|  | Pgl_GLEAN_10031148 |  | -3.32 | -2.19 |  |  |  |
| Ribose-5-phosphate isomerase OS=Setaria | Pgl_GLEAN_10033869 | -2.51 | -4.22 | -3.90 |  |  | 2.65 |
|  | Pgl_GLEAN_10023586 | -1.60 | -2.81 | -2.60 |  |  | 1.64 |

| **Table S13: List of DEGs related to photosynthesis identified in response to –Fe, –Zn, –Fe–Zn stress in leaf and root comparison** | | | | | | | |
| --- | --- | --- | --- | --- | --- | --- | --- |
|  |  |  |  |  |  |  |  |
| **Description** | **GeneIDs** | **Leaf+Fe–Zn Root–Fe+Zn** | **Leaf+Fe–Zn Root–Fe–Zn** | **Leaf–Fe+Zn Root–Fe–Zn** | **Root+Fe–Zn Leaf–Fe+Zn** | **Root+Fe–Zn Leaf–Fe–Zn** | **Root–Fe+Zn Leaf–Fe–Zn** |
| NADPH-protochlorophyllide oxidoreductase | Pgl_GLEAN_10028726 | -3.17 | -8.35 | -7.94 | -1.90 |  | 2.55 |
| Magnesium chelatase OS=Setaria | Pgl_GLEAN_10012641 | -2.54 | -6.36 | -5.84 |  |  | 2.01 |
| Mg-protoporphyrin IX chelatase OS=Setaria | Pgl_GLEAN_10038264 | -2.37 | -4.95 | -4.45 |  |  | 1.84 |
| Chlorophyll a-b binding protein, chloroplastic | Pgl_GLEAN_10015796 | -3.32 | -8.29 | -6.88 |  | 1.51 | 2.82 |
|  | Pgl_GLEAN_10010788 | -2.95 | -9.83 | -9.13 |  | 1.56 | 2.87 |
| Plastoquinol--plastocyanin reductase OS=Setaria | Pgl_GLEAN_10014480 | -2.92 | -6.98 | -6.23 |  |  | 2.66 |
| Plastocyanin OS=Panicum hallii | Pgl_GLEAN_10001189 | -2.98 | -8.49 | -7.74 |  | 1.82 | 2.97 |
| Photosystem II 10 kDa polypeptide, chloroplastic | Pgl_GLEAN_10023088 | -2.90 | -8.56 | -8.24 |  |  | 2.89 |
| Photosystem II CP47 reaction center | Pgl_GLEAN_10001458 |  | -3.64 | -6.64 |  | 3.43 | 3.20 |
| Ferredoxin--NADP reductase, chloroplastic | Pgl_GLEAN_10003660 | -2.32 | -5.83 | -5.12 |  |  | 1.60 |
|  | Pgl_GLEAN_10031758 | -2.66 | -7.32 | -7.13 |  | 1.53 | 3.22 |
| Carbonic anhydrase OS=Setaria | Pgl_GLEAN_10007313 | -2.77 | -5.86 | -4.45 |  | 1.62 | 2.91 |
| Ferrochelatase OS=Setaria viridis | Pgl_GLEAN_10011603 | -1.63 | -3.09 | -3.41 |  |  | 1.81 |
| PSII 6.1 kDa protein OS=Setaria italica | Pgl_GLEAN_10019587 | -2.81 | -7.00 | -7.13 |  |  | 3.02 |
|  | Pgl_GLEAN_10020924 | -3.02 | -8.57 | -8.39 |  |  | 2.91 |
| PSI subunit V OS=Panicum hallii | Pgl_GLEAN_10020246 | -3.03 | -8.45 | -8.16 |  |  | 2.91 |
| Thioredoxin-like 3-1, chloroplastic | Pgl_GLEAN_10031611 | -1.55 | -2.06 | -2.13 |  |  | 1.94 |
| Thioredoxin reductase OS=Setaria italica | Pgl_GLEAN_10022875 | -2.35 | -2.06 | -2.08 | -1.80 |  | 1.54 |
| Thioredoxin H-type 2-like OS=Panicum | Pgl_GLEAN_10005672 | -2.76 | -8.82 | -8.65 |  |  | 2.56 |
| Ferredoxin--NADP reductase, chloroplastic | Pgl_GLEAN_10003660 | -2.32 | -5.83 | -5.12 |  |  | 1.60 |
| Ubiquinol oxidase OS=Setaria viridis | Pgl_GLEAN_10004207 | 3.93 | 5.17 | 3.81 |  |  | -3.31 |
| Sucrose synthase OS=Setaria italica OX=4555 | Pgl_GLEAN_10008200 | 3.02 | 2.99 | 2.93 |  |  | -2.31 |
| Photosystem I P700 chlorophyll a apoprotein | Pgl_GLEAN_10022154 | -1.81 | -6.17 | -6.37 |  | 3.47 | 3.82 |
| Ribulose-phosphate 3-epimerase OS=Setaria | Pgl_GLEAN_10006455 | 2.35 | 1.72 | 1.64 | 1.93 |  | -1.66 |
|  | Pgl_GLEAN_10002011 | -1.70 | -2.66 | -2.39 |  |  | 1.86 |
| Ribulose bisphosphate carboxylase | Pgl_GLEAN_10027288 | -1.79 | -6.06 | -8.09 |  | 3.60 | 3.93 |
|  | Pgl_GLEAN_10020566 | -2.75 | -8.41 | -8.45 |  | 1.54 | 2.64 |
|  | Pgl_GLEAN_10020570 | -2.68 | -6.86 | -6.94 |  | 1.89 | 2.79 |
|  | Pgl_GLEAN_10020572 | -2.74 | -6.67 | -6.88 |  |  | 2.67 |
| Ferredoxin OS=Sorghum bicolor OX=4558 | Pgl_GLEAN_10003612 | -2.77 | -7.83 | -7.63 |  |  | 2.45 |
|  | Pgl_GLEAN_10034138 | -2.77 | -6.27 | -6.17 |  |  | 2.67 |
|  | Pgl_GLEAN_10016441 | -2.77 | -5.81 | -5.43 |  |  | 2.36 |
|  | Pgl_GLEAN_10003613 | -2.81 | -8.31 | -7.67 |  |  | 2.29 |
| Delta-aminolevulinic acid dehydratase | Pgl_GLEAN_10038512 | -1.56 | -1.72 | -1.55 |  |  |  |
| Chlorophyllide a oxygenase chloroplastic | Pgl_GLEAN_10026767 |  | -4.17 | -3.38 |  |  |  |
| NAD(P)H dehydrogenase subunit | Pgl_GLEAN_10013683 | -1.98 | -3.92 | -3.82 |  |  | 2.02 |
| ATP synthase subunit beta OS=Oryza | Pgl_GLEAN_10000054 | 2.24 |  | 2.70 |  |  |  |
| ATP synthase subunit alpha OS=Aegilops | Pgl_GLEAN_10024475 | 2.45 | 1.70 |  |  |  |  |

| **Table S14: List of DEGs related to carbohydrate metabolism identified in response to –Fe, –Zn, –Fe–Zn stress in leaf and root comparison** | | | | | | | |
| --- | --- | --- | --- | --- | --- | --- | --- |
|  |  |  |  |  |  |  |  |
| **Description** | **GeneIDs** | **Leaf+Fe–Zn Root–Fe+Zn** | **Leaf+Fe–Zn Root–Fe–Zn** | **Leaf–Fe+Zn Root–Fe–Zn** | **Root+Fe–Zn Leaf–Fe+Zn** | **Root+Fe–Zn Leaf–Fe–Zn** | **Root–Fe+Zn Leaf–Fe–Zn** |
| Pyruvate, phosphate dikinase OS=Setaria | Pgl_GLEAN_10001175 | -2.68 | -7.34 | -7.37 | -2.10 |  | 2.69 |
| Malate dehydrogenase OS=Setaria italica | Pgl_GLEAN_10015627 | -2.41 | -5.33 | -5.34 | -1.63 |  | 2.10 |
|  | Pgl_GLEAN_10023689 | -2.53 | -5.92 | -5.76 | -1.67 |  | 2.35 |
| D-3-phosphoglycerate dehydrogenase OS=Sorghum | Pgl_GLEAN_10010057 | -2.79 | -5.94 | -5.75 | -2.04 |  | 2.88 |
| Phosphoglycerate kinase OS=Setaria | Pgl_GLEAN_10010879 | -3.04 | -3.66 | -3.79 | -3.01 |  | 3.04 |
| Glyceraldehyde-3-phosphate dehydrogenase | Pgl_GLEAN_10000341 | 3.04 | 3.52 | 2.77 |  |  | -2.73 |
| Pyruvate kinase OS=Setaria italica OX=4555 | Pgl_GLEAN_10004659 | 1.98 | 2.01 | 2.54 |  |  | -1.71 |
| Aldose 1-epimerase OS=Setaria | Pgl_GLEAN_10021973 | 4.60 | 5.42 | 2.82 |  |  | -3.74 |
| Phosphoglycerate mutase (2,3-diphosphoglycerate-dependent) | Pgl_GLEAN_10007450 | 4.13 | 5.33 | 2.42 |  |  | -3.14 |
| Phosphoenolpyruvate carboxylase | Pgl_GLEAN_10037989 | 2.23 | 2.31 | 2.31 |  |  | -2.20 |
| Pyrophosphate--fructose 6-phosphate | Pgl_GLEAN_10011778 | 2.51 | 2.83 | 2.05 |  |  | -2.04 |
| Fructose-bisphosphate aldolase OS=Setaria | Pgl_GLEAN_10038255 | -2.73 | -8.25 | -7.60 |  |  | 2.55 |
| Transketolase OS=Setaria italica OX=4555 | Pgl_GLEAN_10009645 | -1.85 | -2.03 | -2.32 |  |  | 2.41 |
| Phosphoenolpyruvate carboxykinase (ATP) | Pgl_GLEAN_10014472 | 2.17 | 3.70 | 4.02 |  |  | -2.28 |
|  | Pgl_GLEAN_10029931 | -2.45 | -6.91 | -6.39 |  |  | 2.54 |
| Isocitrate dehydrogenase [NADP] OS=Setaria | Pgl_GLEAN_10018367 | -1.96 | -1.55 |  |  |  | -1.66 |
| Nitrate reductase OS=Panicum miliaceum | Pgl_GLEAN_10033153 | -2.16 | -2.39 |  |  |  | 1.95 |
| L-lactate dehydrogenase OS=Setaria italica | Pgl_GLEAN_10018121 |  | 2.45 | 2.97 |  |  |  |

| **Table S15: List of DEGs related to plant hormones biosynthesis identified in response to –Fe, –Zn, –Fe–Zn stress in leaf and root comparison** | | | | | | | |
| --- | --- | --- | --- | --- | --- | --- | --- |
|  |  |  |  |  |  |  |  |
| **Description** | **GeneIDs** | **Leaf+Fe–Zn Root–Fe+Zn** | **Leaf+Fe–Zn Root–Fe–Zn** | **Leaf–Fe+Zn Root–Fe–Zn** | **Root+Fe–Zn Leaf–Fe+Zn** | **Root+Fe–Zn Leaf–Fe–Zn** | **Root–Fe+Zn Leaf–Fe–Zn** |
| Auxin efflux carrier component | Pgl_GLEAN_10017700 | 8.16 | 9.20 | 3.22 |  |  | -4.26 |
| Auxin-responsive protein OS=Setaria italica | Pgl_GLEAN_10017400 | 3.23 | 3.55 | 3.51 | 2.31 |  | -2.50 |
| Cytokinin-N-glucosyltransferase | Pgl_GLEAN_10036480 | 4.39 | 5.47 | 3.81 |  |  | -4.77 |
| Cytokinin dehydrogenase OS=Setaria | Pgl_GLEAN_10036765 | 4.92 | 5.95 | 2.90 |  |  | -2.36 |
| 1-aminocyclopropane-1-carboxylate | Pgl_GLEAN_10016590 | 2.63 | 2.05 | 3.18 | 2.90 |  |  |
| 15-cis-phytoene synthase OS=Sorghum bicolor | Pgl_GLEAN_10005849 | 4.40 | 5.60 | 3.96 |  |  | -4.36 |
| 15-cis-phytoene desaturase, chloroplastic/chromoplastic | Pgl_GLEAN_10008708 | -1.83 |  |  |  |  | 1.91 |
| Auxin-responsive protein SAUR36 | Pgl_GLEAN_10002746 | -2.47 | -6.05 | -5.14 |  | 2.32 | 3.49 |
| Cytokinin riboside 5'-monophosphate phosphoribohydrolase | Pgl_GLEAN_10035660 | 4.26 | 4.54 |  |  |  | -1.92 |
| Cytokinin riboside 5'-monophosphate | Pgl_GLEAN_10016650 | -2.28 | -5.06 | -3.28 |  |  | 1.60 |
| Cytochrome P450 94A1 OS=Dichanthelium | Pgl_GLEAN_10014404 | -2.08 | -2.82 | -2.10 |  |  | 1.82 |
|  | Pgl_GLEAN_10038157 | 5.95 | 6.27 | 3.98 |  |  | -4.51 |
|  | Pgl_GLEAN_10038156 | -2.47 | -3.48 | -3.23 |  | 1.97 | 2.85 |
|  | Pgl_GLEAN_10032881 | -2.97 | -7.67 | -7.64 | -2.11 |  | 2.99 |
|  | Pgl_GLEAN_10023054 | 2.47 | 3.00 | 3.32 |  |  |  |

| **Table S16: List of DEGs related to other cellular pathways identified in response to –Fe, –Zn, –Fe–Zn stress in leaf and root comparison** | | | | | | | |
| --- | --- | --- | --- | --- | --- | --- | --- |
|  |  |  |  |  |  |  |  |
| **Description** | **GeneIDs** | **Leaf+Fe–Zn Root–Fe+Zn** | **Leaf+Fe–Zn Root–Fe–Zn** | **Leaf–Fe+Zn Root–Fe–Zn** | **Root+Fe–Zn Leaf–Fe+Zn** | **Root+Fe–Zn Leaf–Fe–Zn** | **Root–Fe+Zn Leaf–Fe–Zn** |
| Serine/threonine-protein kinase | Pgl_GLEAN_10035380 | 1.94 | 2.50 | 5.76 |  |  |  |
| O-fucosyltransferase family protein | Pgl_GLEAN_10002546 |  | 2.39 | 2.41 |  | -2.01 | -1.72 |
| Patatin OS=Setaria viridis OX=4556 | Pgl_GLEAN_10007971 | 2.34 | 2.57 | 2.31 |  |  | -1.91 |
|  | Pgl_GLEAN_10017277 | 2.32 | 3.19 | 2.28 |  |  | -1.63 |
| Laccase OS=Setaria viridis OX=4556 | Pgl_GLEAN_10000529 | 2.71 | 3.76 | 3.37 |  |  | -2.62 |
|  | Pgl_GLEAN_10004406 | 4.24 | 5.63 | 4.74 |  |  | -5.18 |
|  | Pgl_GLEAN_10014339 | 3.95 | 5.50 | 4.57 |  | -3.34 | -3.21 |
|  | Pgl_GLEAN_10032468 | 4.47 | 4.85 | 4.44 |  | -3.19 | -4.43 |
|  | Pgl_GLEAN_10009302 | 3.40 | 4.07 | 2.85 |  |  | -2.94 |
|  | Pgl_GLEAN_10009305 | 3.12 | 4.25 | 3.50 |  | -1.99 | -2.69 |
|  | Pgl_GLEAN_10006570 | 1.85 | 1.76 |  |  |  |  |
|  | Pgl_GLEAN_10028582 | 7.39 | 8.50 | 4.61 |  | -4.20 | -4.96 |
| Kinesin-like protein OS=Setaria italica | Pgl_GLEAN_10014929 | 6.14 | 7.52 | 3.48 |  |  | -3.03 |
|  | Pgl_GLEAN_10020595 | 7.13 | 8.05 | 4.04 |  |  | -4.28 |
| MLO-like protein OS=Setaria viridis | Pgl_GLEAN_10022428 | -2.07 | -2.47 | -1.82 |  |  | 2.22 |
|  | Pgl_GLEAN_10014660 | 8.15 | 8.82 | 4.82 |  |  | -4.36 |
| NBS-LRR-like protein OS=Cenchrus americanus | Pgl_GLEAN_10024314 | 2.15 | 2.06 | 2.11 |  |  |  |
| Expansin OS=Setaria viridis OX=4556 | Pgl_GLEAN_10021266 | 7.30 | 8.35 | 4.94 |  | -3.42 | -4.71 |
| Germin-like protein OS=Setaria italica | Pgl_GLEAN_10035236 | 6.05 | 5.39 | 5.30 | 4.84 |  | -4.20 |
| Diacylglycerol O-acyltransferase OS=Setaria | Pgl_GLEAN_10021320 | -1.77 | -4.52 | -3.72 |  |  |  |
|  | Pgl_GLEAN_10029766 | -5.17 | -5.43 |  |  |  |  |
|  | Pgl_GLEAN_10004086 | -3.10 | -4.25 | -3.62 | -2.65 |  | 2.80 |
| Trehalose 6-phosphate phosphatase OS=Setaria | Pgl_GLEAN_10026580 | 3.59 | 4.29 |  |  | -3.75 | -4.20 |
|  | Pgl_GLEAN_10028200 | 6.46 | 6.74 |  |  |  |  |
| Peroxidase OS=Setaria viridis | Pgl_GLEAN_10003702 | 8.22 | 8.21 | 5.11 | 2.36 | -3.67 | -5.91 |
| Superoxide dismutase OS=Zea mays | Pgl_GLEAN_10001023 | 2.73 | 3.36 | 1.85 |  |  |  |
|  | Pgl_GLEAN_10037098 | -2.65 | -3.81 | -3.13 | -1.86 |  | 2.16 |
| Serine/threonine protein phosphatase 2A | Pgl_GLEAN_10035997 | -1.65 |  |  |  |  |  |
| Caffeic acid O-methyltransferase | Pgl_GLEAN_10023930 |  | 1.95 | 2.90 |  |  | -2.05 |
| Cinnamoyl-CoA reductase 2-like | Pgl_GLEAN_10017288 |  | 3.87 |  |  |  |  |
| Dirigent protein OS=Triticum aestivum | Pgl_GLEAN_10000800 | -3.49 | -9.42 | -8.02 |  | 1.71 | 3.05 |
| 3-ketoacyl-CoA synthase OS=Setaria italica | Pgl_GLEAN_10030730 | -2.81 | -7.74 | -6.57 |  | 2.47 | 3.67 |
|  | Pgl_GLEAN_10038530 | -1.99 | -5.70 | -3.98 |  | 1.56 | 2.37 |
| Phospholipase A1 OS=Setaria viridis | Pgl_GLEAN_10021604 | -3.11 | -6.27 | -6.10 |  | 2.15 | 3.68 |
|  | Pgl_GLEAN_10024567 | -2.96 | -5.46 | -4.68 | -1.72 |  | 3.23 |
|  | Pgl_GLEAN_10008832 | 6.50 | 6.78 | 3.92 |  |  | -4.77 |
|  | Pgl_GLEAN_10021605 | -3.79 | -6.29 | -4.72 |  | 2.31 | 4.68 |
| Catalase OS=Sorghum bicolor OX=4558 | Pgl_GLEAN_10018073 | -2.34 | -3.19 | -1.95 |  |  | 2.10 |
| L-ascorbate peroxidase OS=Setaria italica | Pgl_GLEAN_10006755 | -2.37 | -4.83 | -4.30 |  |  | 2.04 |

**Table S17: Important genes associated with the Fe and Zn uptake, transport in the leaf and root tissues of pearl millet.**

| **Gene ID** | **Seq Description** | **Gene ontology (GO)** |
| --- | --- | --- |
| Pgl_GLEAN_10012139 | 2'-deoxymugineic-acid 2'-dioxygenase | Dioxygenase activity Metal ion binding |
| Pgl_GLEAN_10016462 | 5-methyltetrahydropteroyltriglutamate--homocysteine | Methionine biosynthetic process |
| Pgl_GLEAN_10004517 | 5-methyltetrahydropteroyltriglutamate--homocysteine | Methionine biosynthetic process |
| Pgl_GLEAN_10014564 | Adenine phosphoribosyltransferase | Adenine phosphoribosyltransferase activity Adenine salvage pathway |
| Pgl_GLEAN_10012911 | Adenine phosphoribosyltransferase | Adenine phosphoribosyltransferase activity Adenine salvage pathway |
| Pgl_GLEAN_10029973 | Adenine phosphoribosyltransferase | Adenine phosphoribosyltransferase activity Adenine salvage pathway |
| Pgl_GLEAN_10024237 | Aldo_ket_red domain-containing | 3''-deamino-3''-oxonicotianamine reductase activity  Cellular response to zinc ion starvation  Response to iron ion starvation  Siderophore biosynthetic process |
| Pgl_GLEAN_10036166 | Calcium-transporting ATPase | ATPase-coupled cation transmembrane transporter activity |
| Pgl_GLEAN_10036492 | Cation/H(+) antiporter 14 | Monovalent inorganic cation transport |
| Pgl_GLEAN_10018243 | Cation-chloride cotransporter | Integral component of membrane Transmembrane transport |
| Pgl_GLEAN_10019693 | Formate dehydrogenase, mitochondrial | Formate dehydrogenase (NAD+) activity |
| Pgl_GLEAN_10002079 | Ferritin | Iron ion binding  Intracellular sequestering of iron ion  Iron ion transport |
| Pgl_GLEAN_10008667 | Heavy metal transport/detoxification | Metal ion transport |
| Pgl_GLEAN_10020996 | Mugineic-acid 3-dioxygenase | 2'-deoxymugineic-acid 3-dioxygenase activity  Mugineic-acid 3-dioxygenase activity |
| Pgl_GLEAN_10016104 | Oligopeptide transporter 3 | Iron ion homeostasis Xylem-to-phloem iron transport |
| Pgl_GLEAN_10030576 | HMA domain-containing protein | Metal ion transport |
| Pgl_GLEAN_10006197 | HMA domain-containing protein | Metal ion transport |
| Pgl_GLEAN_10008446 | Plasma membrane ATPase | Proton-exporting ATPase activity  Proton export across plasma membrane |
| Pgl_GLEAN_10014811 | Plasma membrane ATPase | Proton-exporting ATPase activity  Proton export across plasma membrane |
| Pgl_GLEAN_10005234 | Plasma membrane ATPase | Proton-exporting ATPase activity  Proton export across plasma membrane |
| Pgl_GLEAN_10036818 | Probable O-methyltransferase 2 | O-methyltransferase activity  S-adenosylmethionine-dependent methyltransferase activity |
| Pgl_GLEAN_10014045 | Probable O-methyltransferase 2 | O-methyltransferase activity S-adenosylmethionine-dependent methyltransferase activity |
| Pgl_GLEAN_10019136 | Probable O-methyltransferase 2 | O-methyltransferase activity S-adenosylmethionine-dependent methyltransferase activity |
| Pgl_GLEAN_10025753 | Protein ZINC INDUCED FACILITATOR-LIKE | Integral component of membrane  Transmembrane transporter activity |
| Pgl_GLEAN_10026234 | Solute carrier family 40 protein | Iron ion transmembrane transporter activity |
| Pgl_GLEAN_10030183 | Solute carrier family 40 protein | Iron ion transmembrane transporter activity |
| Pgl_GLEAN_10004868 | Solute carrier family 40 protein | Iron ion transmembrane transporter activity  Iron ion transport |
| Pgl_GLEAN_10033886 | Transcription factor FER-LIKE | Protein dimerization activity |
| Pgl_GLEAN_10033596 | Uncharacterized protein | Zinc ion transmembrane transporter activity |
| Pgl_GLEAN_10026643 | Uncharacterized protein | Zinc ion transmembrane transporter activity |
| Pgl_GLEAN_10012759 | Uncharacterized protein | Zinc ion transmembrane transporter activity |
| Pgl_GLEAN_10003646 | Uncharacterized protein | Iron-nicotianamine transmembrane transporter activity Response to iron ion Seed development |
| Pgl_GLEAN_10015686 | Uncharacterized protein | Nicotianamine synthase activity  Nicotianamine biosynthetic process |
| Pgl_GLEAN_10023362 | Uncharacterized protein | O-methyltransferase activity |
| Pgl_GLEAN_10034360 | Uncharacterized protein | Zinc ion transmembrane transporter activity  Zinc ion transmembrane transport |
| Pgl_GLEAN_10030145 | Uncharacterized protein | Nicotianamine synthase activity  Nicotianamine biosynthetic process |
| Pgl_GLEAN_10021386 | Uncharacterized protein | Iron ion homeostasis |
| Pgl_GLEAN_10001420 | Uncharacterized protein | Metal ion transmembrane transporter activity  Zinc ion transport |
| Pgl_GLEAN_10023361 | Uncharacterized protein | O-methyltransferase activity  S-adenosylmethionine-dependent methyltransferase activity |
| Pgl_GLEAN_10026644 | Uncharacterized protein | Zinc ion transmembrane transporter activity  Zinc ion transmembrane transport |
| Pgl_GLEAN_10025175 | Uncharacterized protein | O-methyltransferase activity  S-adenosylmethionine-dependent methyltransferase activity |
| Pgl_GLEAN_10005019 | Uncharacterized protein | Iron-nicotianamine transmembrane transporter activity Response to iron ion Seed development |
| Pgl_GLEAN_10004175 | Uncharacterized protein | O-methyltransferase activity  S-adenosylmethionine-dependent methyltransferase activity |
| Pgl_GLEAN_10012625 | Uncharacterized protein | O-methyltransferase activity  S-adenosylmethionine-dependent methyltransferase activity |
| Pgl_GLEAN_10037076 | Uncharacterized protein | O-methyltransferase activity  S-adenosylmethionine-dependent methyltransferase activity |
| Pgl_GLEAN_10008622 | Uncharacterized protein | O-methyltransferase activity  S-adenosylmethionine-dependent methyltransferase activity |
| Pgl_GLEAN_10029601 | Uncharacterized protein | O-methyltransferase activity  S-adenosylmethionine-dependent methyltransferase activity |
| Pgl_GLEAN_10038143 | Uncharacterized protein | O-methyltransferase activity  S-adenosylmethionine-dependent methyltransferase activity |
| Pgl_GLEAN_10013687 | Uncharacterized protein | Iron ion transmembrane transporter activity  Intracellular sequestering of iron ion |
| Pgl_GLEAN_10019761 | Uncharacterized protein | Zinc ion transmembrane transporter activity  Zinc ion transmembrane transport |
| Pgl_GLEAN_10028548 | Uncharacterized protein | O-methyltransferase activity  S-adenosylmethionine-dependent methyltransferase activity |
| Pgl_GLEAN_10005377 | Uncharacterized protein | Zinc ion transmembrane transporter activity  Zinc ion transmembrane transport |
| Pgl_GLEAN_10019232 | Uncharacterized protein | S-adenosyl-L-methionine transmembrane transporter activity |
| Pgl_GLEAN_10034895 | Vacuolar cation/proton exchanger | Plant-type vacuole membrane  Calcium:proton antiporter activity  Calcium ion transmembrane transport |
| Pgl_GLEAN_10036960 | Vacuolar cation/proton exchanger | Plant-type vacuole membrane  Calcium:proton antiporter activity  Calcium ion transmembrane transport |
| Pgl_GLEAN_10032199 | Zinc transporter 3 | Zinc ion transmembrane transporter activity |
| Pgl_GLEAN_10036448 | Zinc transporter 5 | Zinc ion transmembrane transporter activity |
| Pgl_GLEAN_10006489 | S-adenosylmethionine synthase | Methionine adenosyltransferase activity  S-adenosylmethionine biosynthetic process |
| Pgl_GLEAN_10014808 | Uncharacterized protein | Response to zinc ion |
| Pgl_GLEAN_10023586 | Ribose-5-phosphate isomerase | Ribose-5-phosphate isomerase activity  Pentose-phosphate shunt, non-oxidative branch |
| Pgl_GLEAN_10033869 | Ribose-5-phosphate isomerase | Ribose-5-phosphate isomerase activity  Pentose-phosphate shunt, non-oxidative branch |
| Pgl_GLEAN_10007363 | ABC transporter B family member | ATPase-coupled transmembrane transporter activity |
| Pgl_GLEAN_10036364 | ABC transporter B family member | ATPase-coupled transmembrane transporter activity |
| Pgl_GLEAN_10019636 | ABC transporter F family member 1 | ATP binding  ATPase activity |
| Pgl_GLEAN_10020099 | ABC transporter G family member | ATPase-coupled transmembrane transporter activity |
| Pgl_GLEAN_10032979 | ABC transporter G family member | ATPase-coupled transmembrane transporter activity |
| Pgl_GLEAN_10023174 | ABC transporter G family member | ATP binding  ATPase activity |
| Pgl_GLEAN_10038575 | 1,2-dihydroxy-3-keto-5-methylthiopentene | 1,2-dihydroxy-3-keto-5-methylthiopentene activity |

**Figure S1: Validation of selected genes from different pathways expressed in RNAseq through qRT-PCR analysis.**


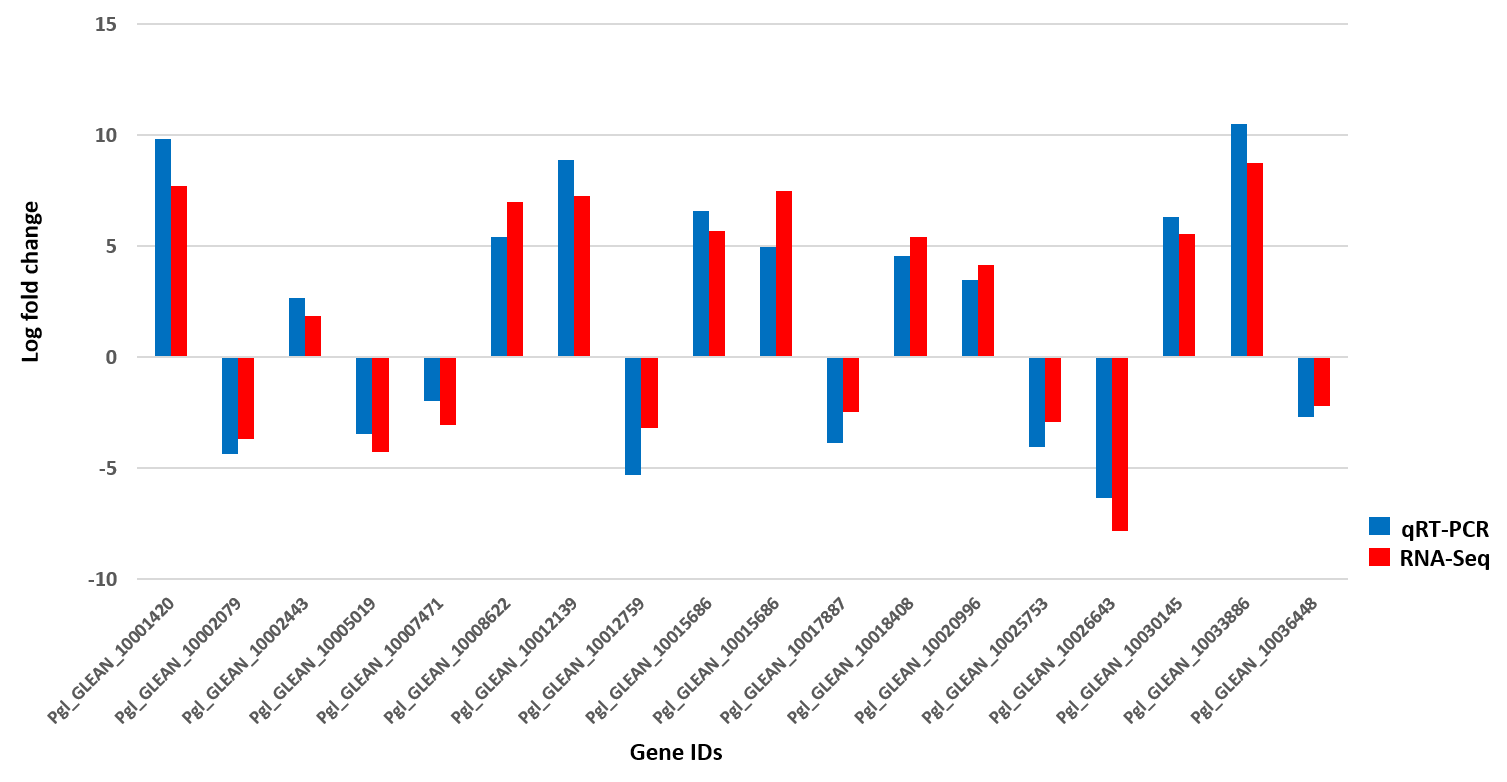


**Figure S2: Heat map of differentially expressed unannotated genes in Fe and Zn stress under 18 pairwise combinations
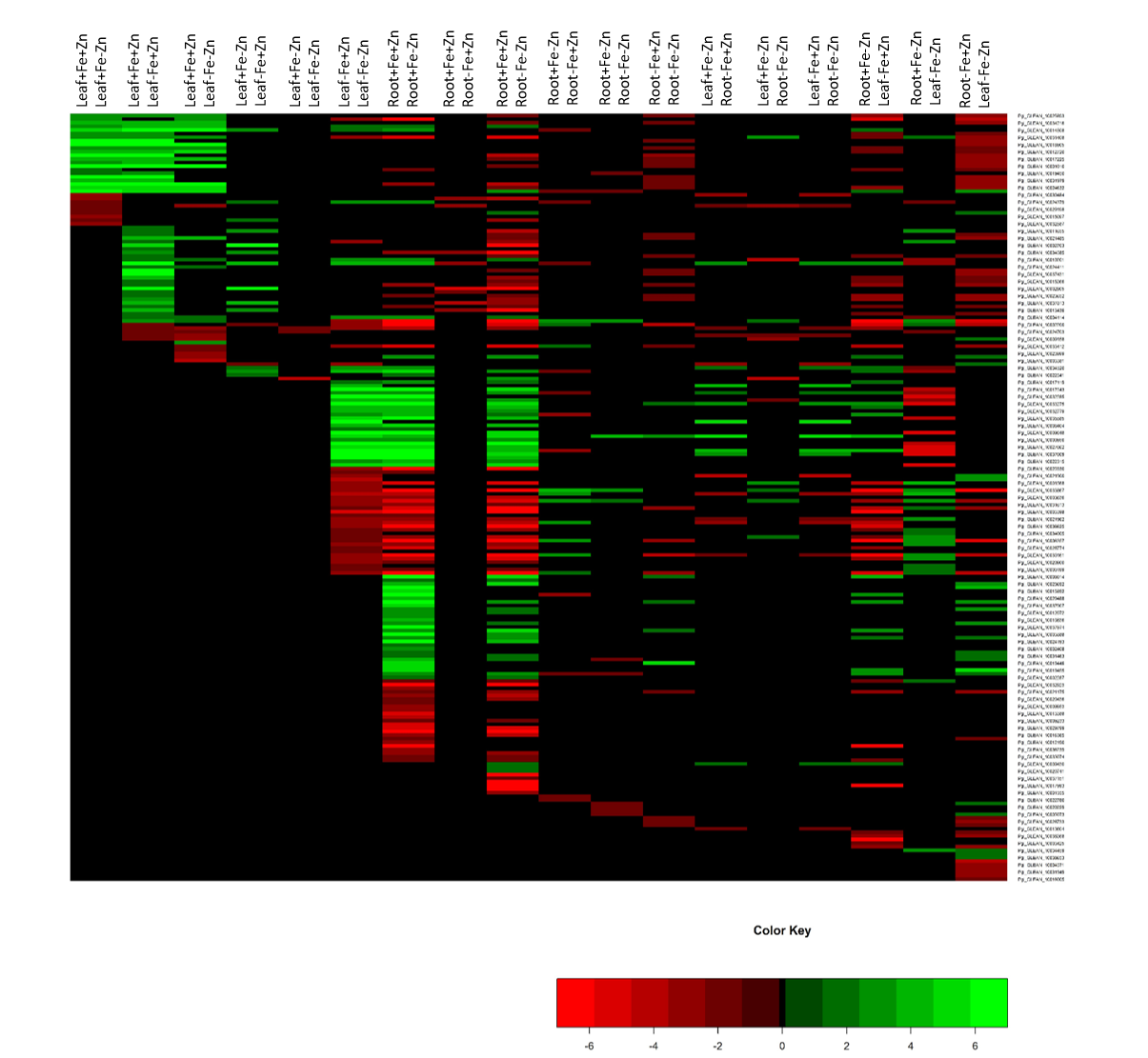
**

**Figure S3: Functional annotations of DEGs under Fe and Zn stress in A) leaf, and B) root. The graphical picture represented the most significant terms under each category viz., biological process (pink), cellular component (green), molecular function (blue) and C) Distribution of biological pathways to the identified DEGs under Fe and Zn stress conditions.**

**
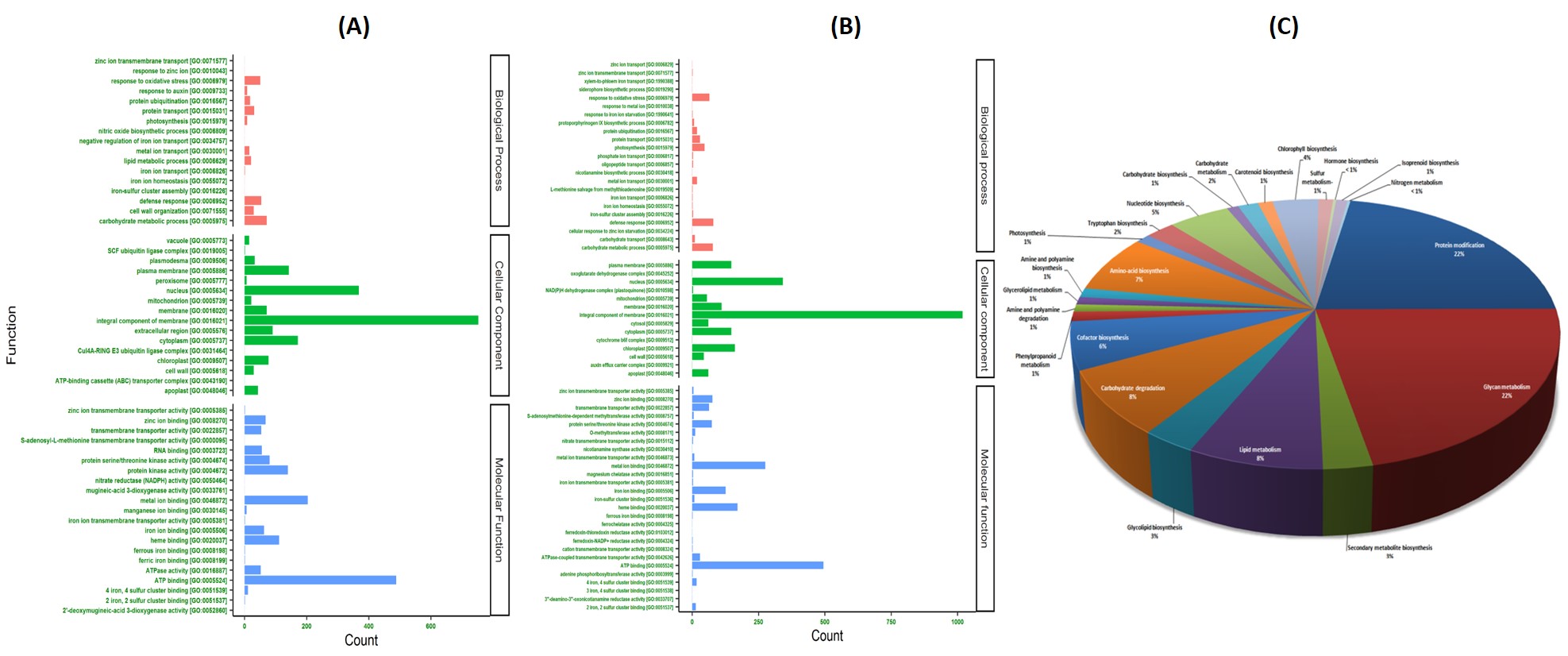
**
